# Scrutiny of NolA and NodD1 Regulatory Roles in Symbiotic Compatibility Unveils New Insights into Bradyrhizobium guangxiense CCBAU53363 Interacting with Peanut (Arachis hypogaea) and Mung Bean (Vigna radiata)

**DOI:** 10.1128/spectrum.02096-22

**Published:** 2022-12-08

**Authors:** Jiao Ying Shang, Pan Zhang, Yu Wen Jia, Yi Ning Lu, Yue Wu, Shuang Ji, La Chen, En Tao Wang, Wen Xin Chen, Xin Hua Sui

**Affiliations:** a State Key Laboratory of Agrobiotechnology, MOA Key Laboratory of Soil Microbiology, College of Biological Sciences, China Agricultural University, Beijing, China; b CAS Key Laboratory of Quantitative Engineering Biology, Shenzhen Institute of Synthetic Biology, Shenzhen Institute of Advanced Technology, Chinese Academy of Sciences, Shenzhen, China; c Escuela Nacional de Ciencias Biológicas, Instituto Politécnico Nacional, Mexico City, México; Integrative Microbiology Research Centre

**Keywords:** peanut bradyrhizobia, NolA, NodD1, nodulation, compatibility

## Abstract

Bradyrhizobium guangxiense CCBAU53363 efficiently nodulates peanut but exhibits incompatible interaction with mung bean. By comparing the common *nod* region with those of other peanut bradyrhizobia efficiently nodulating these two hosts, distinctive characteristics with a single *nodD* isoform (*nodD1*) and a truncated *nolA* were identified. However, the regulatory roles of NodD1 and NolA and their coordination in legume-bradyrhizobial interactions remain largely unknown in terms of explaining the contrasting symbiotic compatibility. Here, we report that *nolA* was important for CCBAU53363 symbiosis with peanut but restricted nodulation on mung bean, while *nodD1* was dispensable for CCBAU53363 symbiosis with peanut but essential for nodulation on mung bean. Moreover, *nolA* exerted a cumulative contribution with *nodD1* to efficient symbiosis with peanut. Additionally, mutants lacking *nolA* delayed nodulation on peanut, and both *nolA* and *nodD1* were required for competitive nodule colonization. It is noteworth that most of the nodulation genes and type III secretion system (T3SS)-related genes were significantly downregulated in a strain 53Δ*nodD1nolA* mutant compared to wild-type strain CCBAU53363, and the downregulated nodulation genes also had a greater impact than T3SS-related genes on the symbiotic defect of 53Δ*nodD1nolA* on peanut, which was supported by a more severe symbiotic defect induced by 53Δ*nodC* than that with the 53Δ*nodD1nopP*, 53Δ*nodD1rhcJ*, and 53Δ*nodD1ttsI* mutants. NolA did not regulate *nod* gene expression but did regulate the T3SS effector gene *nopP* in an indirect way. Meanwhile, *nolA*, *nodW*, and some T3SS-related genes besides *nopP* were also demonstrated as new “repressors” that seriously impaired CCBAU53363 symbiosis with mung bean. Taken together, the roles and essentiality of *nolA* and *nodD1* in modulating symbiotic compatibility are sophisticated and host dependent.

**IMPORTANCE** The main findings of this study were that we clarified that the roles and essentiality of *nodD1* and *nolA* are host dependent. Importantly, for the first time, NolA was found to positively regulate T3SS effector gene *nopP* to mediate incompatibility on mung bean. Additionally, NolA does not regulate *nod* genes, which are activated by NodD1. *nolA* exerts a cumulative effect with *nodD1* on CCBAU53363 symbiosis with peanut. These findings shed new light on our understanding of coordinated regulation of NodD1 and NolA in peanut bradyrhizobia with different hosts.

## INTRODUCTION

Rhizobia induce nodule formation on legume roots or stems and reduce atmospheric nitrogen to ammonium in exchange for carbon sources and essential nutrients from legumes (1). Symbiotic compatibility that usually occurs at the early stage of interaction determines the specificity between the symbiotic partners (2, 3). Meanwhile, it also occurs at the later stage of root nodule development, resulting in the variation of symbiotic nitrogen fixation efficiency (4, 5). To initiate symbiotic process, flavonoids secreted by legumes induce expression of rhizobial nodulation genes (*nod*, *noe*, and *nol*) necessary for synthesis of nodulation factors (NFs), which in turn affect symbiosis performance via its structural change, synthesis, or secretion level (6, 7). Thus, effective symbiosis requires the exact regulation of nodulation gene expression, which is primarily mediated by NodD proteins through interactiion specifically with different flavonoids (8). Copy numbers of *nodD* genes vary from one (9, 10) to five (11, 12) in different rhizobial species, which may contribute to expanding rhizobial host range and circumventing abiotic stresses (12). Generally, *nod* genes are activated by NodD1, while NodD2 regulates them positively or negatively in different rhizobial strains (11, 13). Mutations of *nodD* genes might be dispensable or cause different degrees of impaired symbiotic abilities, depending on different legume-rhizobium partners. For example, a *nodD1* mutant of Sinorhizobium fredii USDA191 failed to nodulate soybean (Glycine max), while *nodD2* inactivation resulted in delayed nodulation or reduced nodule number, depending on the soybean cultivars (14). However, in the well-known soybean symbiont Bradyrhizobium diazoefficiens USDA110, mutants lacking *nodD1* and/or *nodD2* can still nodulate their hosts due to the presence of another activator, NodW, which is critical for activation of *nodD1* and *nod* genes (15–17). Moreover, *nodW* deletion exerted severe nodulation-deficient performance with no nodule formation on mung bean (Vigna radiata) and siratro (Macroptilium atropurpureum) but was dispensable for efficient nodulation on soybean, and only a *nodD1nodW* double mutant virtually lost nodulation capability on soybean (16). Additionally, another transcriptional activator, NolA, in USDA110 activates itself and NodD2, which in turn represses the expression of *nodABCSUIJ* operon (13). Soybean inoculated with *nolA* mutant of USDA110 showed only a slight delay in nodulation, while cowpea (Vigna unguiculata) inoculated with this mutant exhibited serious defects in nodulation and nitrogen fixation (18). Meanwhile, complementation of a *nodD1 nodD2 nolA* triple mutant with *nolA* also could relieve its nodulation deficiencies on soybean to a significant extent, indicating an accumulative contribution of *nolA* and *nodD* to the nodulation process (19). Therefore, the symbiotic adaptation of USDA110 is coordinately controlled by multiple regulators.

As a widespread oilseed and food legume of great agricultural and economic importance, peanut (Arachis hypogaea) can form root nodules with a wide range of *Bradyrhizobium* species (20, 21), which infect peanut roots via epidermal cracks without formation of intracellular infection threads (22). This symbiotic interaction is generally NF dependent, and NFs modulate reactive oxygen species (ROS) by enhancing peanut antioxidant machinery in favor of symbiosis development (23, 24). However, some bradyrhizobia also formed efficient or inefficient nodules on peanut roots in an NF-independent manner (25, 26). Thus, peanut-bradyrhizobium interaction might differ substantially from those of well-known soybean-bradyrhizobium symbiosis (13), and the symbiotic mechanisms underlying the interaction of different peanut bradyrhizobia with their hosts have been largely unexplored so far. Moreover, few studies have focused on how expression of the *nod* operon is coordinately controlled by multiple regulators, such as NodD and NolA in peanut bradyrhizobia, to mediate symbiotic interaction. A scarce case was reported in peanut symbiont *Bradyrhizobium* sp. strain NC92, where *nolA* inactivation rendered delayed nodulation and decreased nodule numbers on siratro and cowpea, while NolA was dispensable for *nod* gene expression (27). Intriguingly, NodD1 repressed *nod* gene expression without induction by the peanut symbiotic signal genistein but did not regulate *nod* expression under the genistein-induced condition (27).

Mung bean is another important functional food crop (28) belonging to the same cross-nodulation group with peanut (29). Thus, peanut bradyrhizobia are generally believed to invade mung bean via root hair curling, followed by the formation and progression of the infection thread (30). However, previously, 30 representative peanut bradyrhizobium strains, collected from three major peanut-producing areas in China, were divided into two nodulation genotypes based on their symbiotic performance and genomic analyses (31). Type I strains, harboring two copies of *nodD* and a full-length *nolA* gene, formed effective nodules with both peanut and mung bean, while type II strains, carrying a single *nodD* (*nodD1*) and a truncated *nolA* gene, induced “supernodulation” with peanut but exhibited incompatible symbiosis with mung bean (31). Due to the host-dependent variation in the symbiotic compatibility for the type II bradyrhizobia, we hypothesized that the regulatory *nodD1* and *nolA* genes as important symbiosis-related transcriptional regulators might be involved in rhizobial adaptations to different hosts. Moreover, it remains largely unknown how NolA regulates symbiotic compatibility in addition to regulating the *nod* operon in *Bradyrhizobium* species and its involvement in coordinated regulation of NodD1 and NolA in symbiotic compatibility.

Considering all of the information mentioned above, the type II representative strain Bradyrhizobium guangxiense CCBAU53363 was chosen to investigate the regulatory roles of *nodD1* and *nolA* in symbiotic adaptation to peanut and mung bean. We first characterized potential roles of *nodD1* and *nolA* alone and then characterized their joint effects in symbiotic compatibility, and the contrasting roles of *nodD1* and/or *nolA* on nodulation and nitrogen fixation of CCBAU53363 on these two hosts were revealed. Then NolA or NodD1-NolA regulons, including nodulation- and type III secretion system (T3SS)-related genes probably accounting for these intriguingly different symbiotic adaptations, were identified by transcriptomic analyses and quantitative real-time PCR (qRT-PCR) experiments with induction by genistein, and their symbiotic interactions were further characterized using individual or combined mutants of nodulation- and T3SS-related genes. Findings obtained in this study highlighted the importance of NodD1, NolA, or their coordination in optimizing symbiotic adaptation mediated by NF and T3SS pathway regulation cascades.

## RESULTS

### Coordinated regulation roles of *nolA* and *nodD1* in CCBAU53363-peanut symbiosis.

Bioinformatic analysis of the CCBAU53363 genome revealed the presence of only one *nodD* gene and a truncated *nolA* gene (417 bp), differing from the other bradyrhizobia, such as *B. diazoefficiens* USDA110, *B. elkanii* USDA61, *B. zhanjiangense* CCBAU51778, and *Bradyrhizobium* sp. strain ORS285, possessing two copies of *nodD* and a full-length version of *nolA* (714 to 738 bp) (see Fig. S1A in the supplemental material). Phylogenetic analysis showed that NodD of CCBAU53363 is a divergent lineage distantly clustered with NodD1 of ORS285, which is supported by an intermediate bootstrap value (66%) (Fig. S1B), and presents the highest similarity of 64.01% to the NodD1 of CCBAU51778. Hence, we supposed it more probably has some regulatory features shared with NodD1 of other rhizobia and designated it NodD1. Additionally, the *nolA*_53363_ gene orientation was opposed to *nodD1*_53363_, in contrast to those in other bradyrhizobia (Fig. S1A). The truncated NolA_53363_ largely matched partial NolA proteins tested (Fig. S2), and the local alignment (amino acids [aa] 14 to 129) showed similarity reaching 60.34% that of USDA61 or NC92, although the similarity of the full alignment is about 32.00% (Table S1).

**FIG 1:**
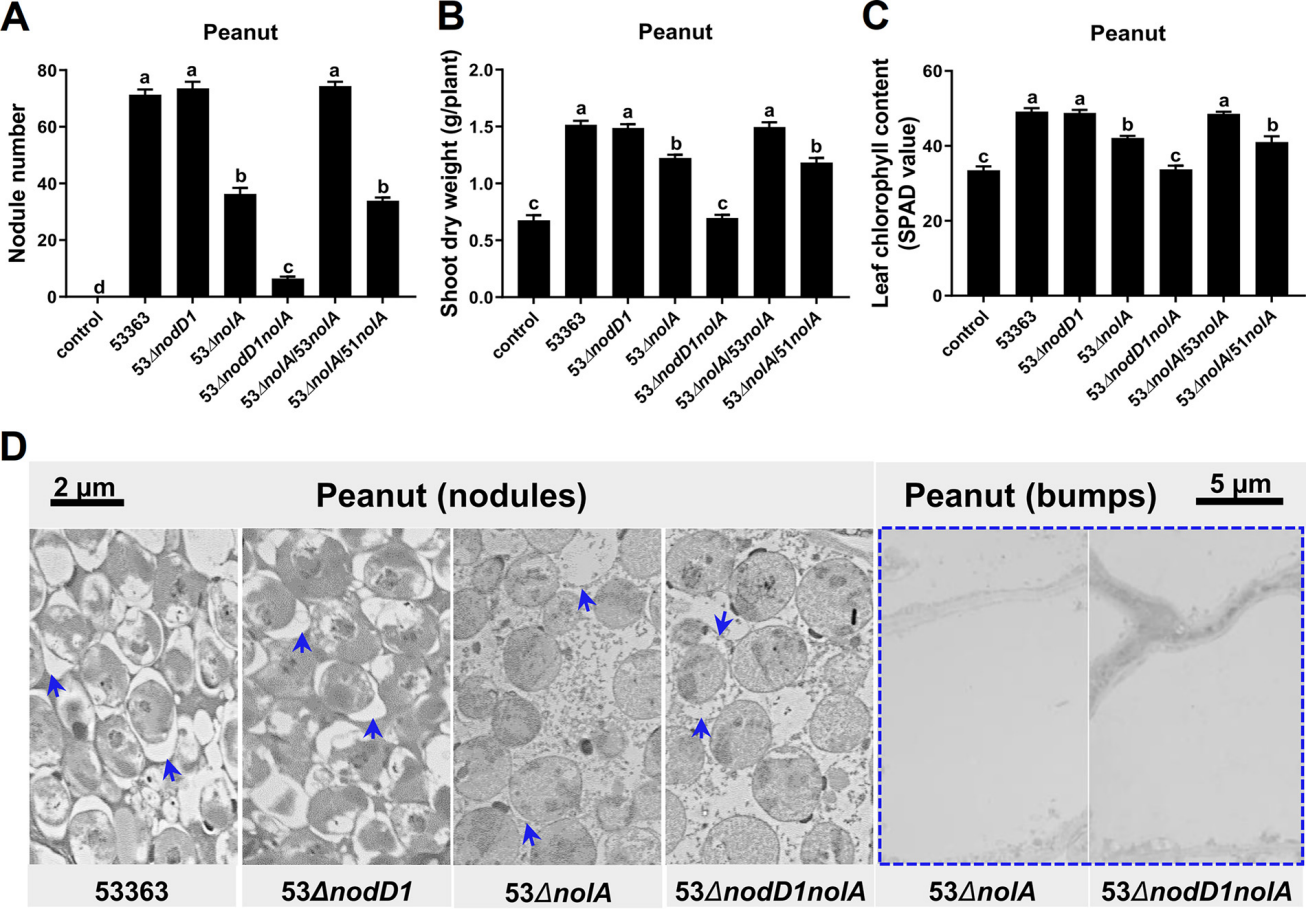
Symbiotic performance of peanut inoculated with CCBAU53363 and mutants lacking *nolA* and/or *nodD1*. (A to C) Number of infected nodules (A), shoot dry weight (B), and leaf chlorophyll content (C) of peanut inoculated with wild-type strain CCBAU53363 or its derivate strains. The values shown are means ± standard errors of the means (SEM) of values from more than 23 plants scored from three independent experiments (7 to 8 replicate plants per treatment). Different lowercase letters indicate significant differences among means based on Duncan’s test (α = 0.05). (D) Transmission electron micrographs of ultrathin sections of infected nodules and bumps (blue border) on peanut inoculated with wild-type strain CCBAU53363 or its derivates. The symbiosome membranes were indicated by blue arrows. Bumps were not infected by rhizobia. The scale bar indicates 2 or 5 μm.

To characterize the roles of *nolA* and *nodD1* in symbiosis on peanut, single- and double-knockout mutants (53Δ*nolA*, 53Δ*nodD1*, and 53Δ*nodD1nolA*) were obtained and inoculated on peanut. The number of nodules induced by 53Δ*nolA* was decreased about 50% compared to that of CCBAU53363 (Fig. 1A), and the shoot dry weight and leaf chlorophyll content were also significantly lower for plants inoculated with 53Δ*nolA* than those inoculated with CCBAU53363 (Fig. 1B and C), while symbiotic performance levels were similar between 53Δ*nodD1* and CCBAU53363 (Fig. 1A to C). Notably, 53Δ*nodD1nolA* showed more severe symbiotic defects on peanut than 53Δ*nolA* (Fig. 1A to C). Compared with the bacteroid number in peanut nodules induced by CCBAU53363, no significant difference existed in 53Δ*nodD1*, the bacteroid number in 53Δ*nolA*- or 53Δ*nodD1nolA*-induced nodules was obviously decreased, and the symbiosome membrane had a seriously damaged appearance with nearly total disappearance (Fig. 1D). It is noteworthy that 53Δ*nolA* and 53Δ*nodD1nolA* also induced many uninfected abnormal bumps on roots, and more abnormal bumps were induced by 53Δ*nodD1nolA* than by 53Δ*nolA* (Fig. S3A), and no infected rhizobia were observed in these bumps (Fig. 1D), implying the potential disturbance of symbiotic signaling. A further complementary experiment revealed that the *nolA*_53363_ gene could complement symbiotic defects of 53Δ*nolA* and root bumps also disappeared, but the heterologous complement *nolA* gene from CCBAU51778, a representative stain of type I peanut bradyrhizobia, did not restore the symbiotic performance (Fig. 1A to C and Fig. S3A). Taken together, *nolA* but not *nodD1* is important for CCBAU53363-peanut symbiosis, while *nolA* cumulatively contributes with *nodD1* to this symbiotic adaptation.

### Negative role of *nolA* and essential role of *nodD1* in nodulation of CCBAU53363 with mung bean.

**FIG 2:**
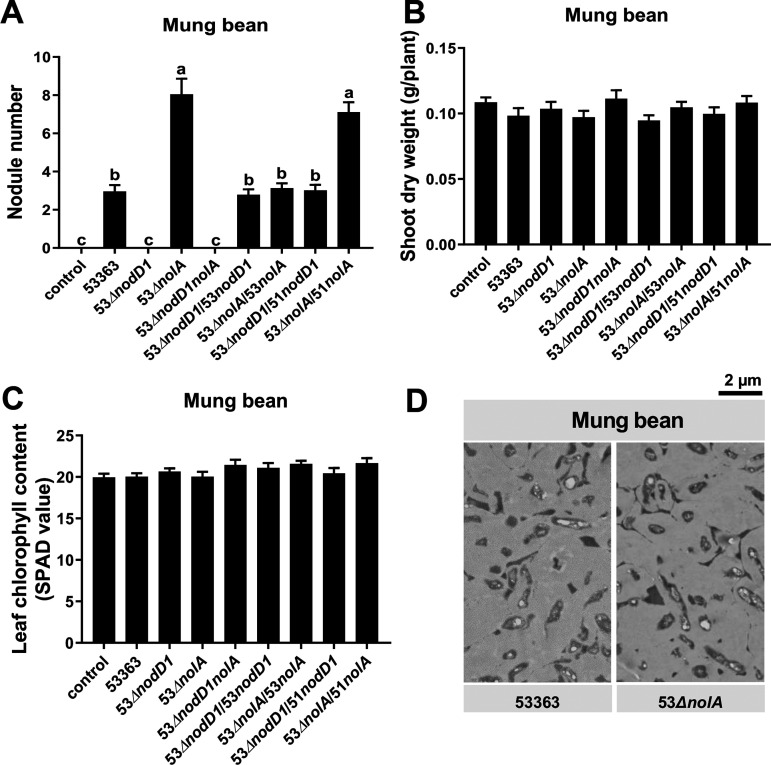
Symbiotic phenotype of CCBAU53363 mutants lacking *nolA* and/or *nodD1* on mung bean. (A to C) Number of infected nodules (A), shoot dry weight (B), and leaf chlorophyll content (C) of mung bean inoculated with wild-type strain CCBAU53363 or its derivate strains. Values shown are means ± SEM of values from more than 27 plants scored from three independent experiments (8 to 10 replicate plants per treatment). Different lowercase letters indicate significant differences among means based on Duncan’s test (α = 0.05). (D) Transmission electron micrographs of ultrathin sections of infected nodules on mung bean inoculated with wild-type strain CCBAU53363 or 53Δ*nolA*. CCBAU53363 mutants lacking *nodD1* could not nodulate mung bean. The scale bar indicates 2 μm.

The contrasting symbiotic phenotypes of CCBAU53363 on peanut and mung bean reminded us to further explore the potential roles of *nolA* and *nodD1* on symbiotic adaptation on mung bean. Interestingly, both 53Δ*nodD1* and 53Δ*nodD1nolA* failed to nodulate mung bean (Fig. 2A), while 53Δ*nolA* induced more and smaller nodules compared to CCBAU53363 (Fig. 2A and Fig. S3B), but the shoot dry weight and leaf chlorophyll content of plants inoculated with CCBAU53363 and 53Δ*nolA* were similar (Fig. 2B and C), indicating a remaining nitrogen fixation defect of 53Δ*nolA*. Moreover, unlike the pink nodules induced by CCBAU53363, the nodules induced by 53Δ*nolA* were yellow-green (Fig. S3B), which might be due to early senescence (32). There was no noticeable difference in the bacteroid number within mung bean nodules induced by 53Δ*nolA* compared to CCBAU53363 (Fig. 2D). Complementary experiments revealed that *nodD1* and *nolA* from CCBAU53363 could, respectively, recover symbiotic performances of 53Δ*nodD1* and 53Δ*nolA* to the wild-type level (Fig. 2A and Fig. S3B). Meanwhile, *nodD1* instead of *nolA* from CCBAU51778 could also complement the nodulation capability of the corresponding mutant on mung bean (Fig. 2A and Fig. S3B). These results suggested that NolA acts as a negative regulator and NodD1 is essential for CCBAU53363 nodulation of mung bean, which is opposed to that in the CCBAU53363-peanut interaction, demonstrating a host-dependent regulatory mode.

### Nodulation delay on peanut with 53Δ*nolA* and *nodD1/nolA* contributing to competitive nodule colonization of CCBAU53363.

**FIG 3:**
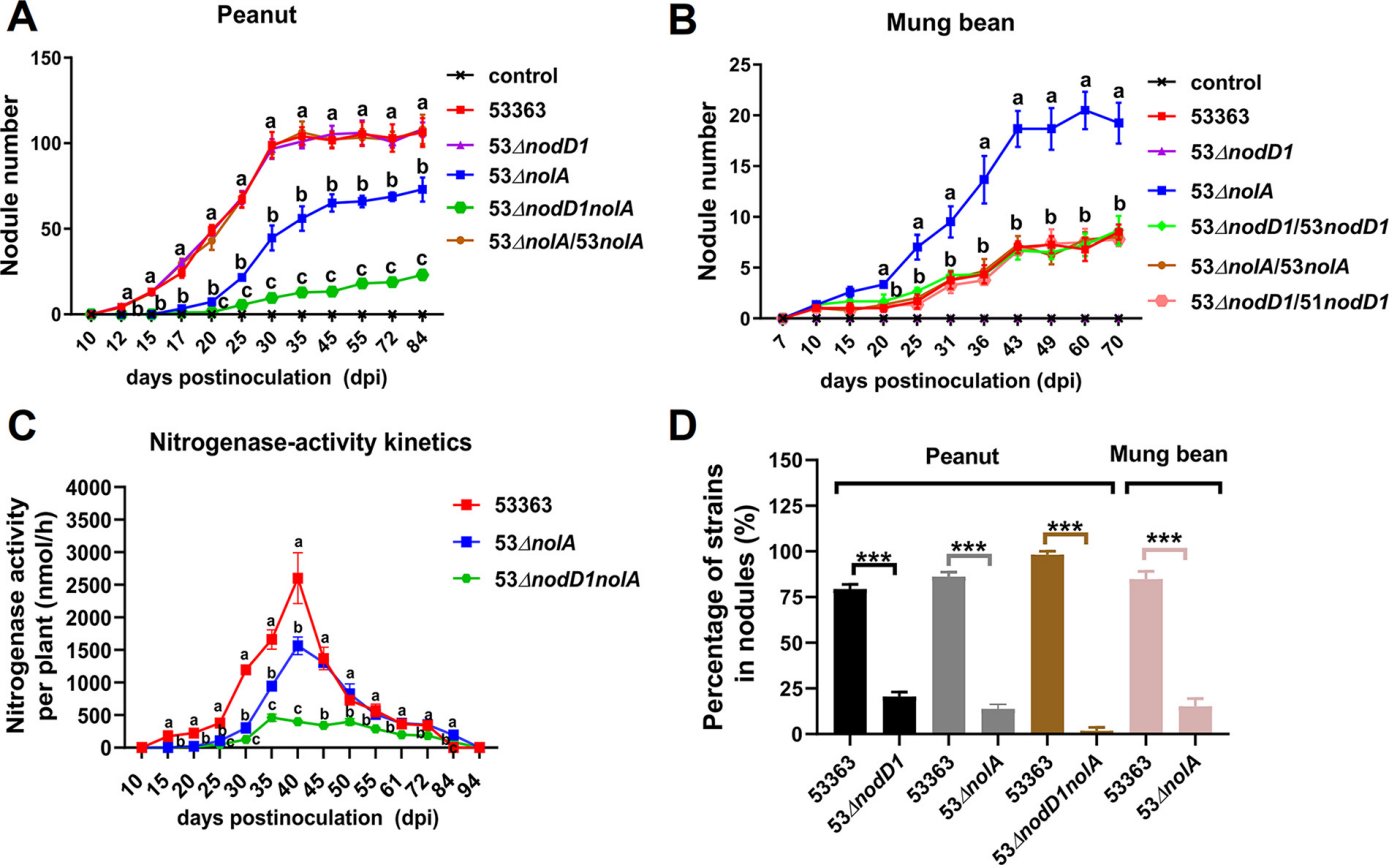
Roles of *nolA* and *nodD1* in nodulation initiation, nitrogenase activity, and competitive nodulation ability. (A and B) Nodule number per peanut plant (A) and nodule number per mung bean plant (B) inoculated with wild-type strain CCBAU53363 or its mutants in periods of dynamic observation. (C) Nitrogenase activity levels per plant of peanut in period of 94 days after inoculation with wild-type strain CCBAU53363 or its derivates. Values shown in panels A to C are means ± SEM from more than 10 plants scored from three independent experiments (3 to 5 replicate plants per treatment). Different lowercase letters indicate significant differences among means based on Duncan’s test (α = 0.05). (D) Relative competitive abilities of wild-type strain CCBAU53363 and derivate mutants. Nodules were collected on peanut and mung bean at 45 and 30 dpi, respectively. The percentage of nodules occupied by each strain was determined by PCR verification of corresponding genes mutated, and the proportion of each reciprocal pair of strains was calculated. Values shown are means ± SEM of nodules scored (601 nodules on peanut and 137 nodules on mung bean) from three independent experiments (3 to 5 replicate plants per treatment). Mutants lacking *nodD1* completely lost the ability to nodulate mung bean, so the competitive nodulation ability of related mutants on mung bean was not determined. The asterisks indicate significant difference between the reciprocal pairs of strains with same bar color (*t* test; ***, *P* < 0.001).

The different impacts of *nolA* and *nodD1* on nodulation speed or competitive nodulation ability have been widely identified (33–36); thus, the nodulation kinetics and nodule occupancy of 53Δ*nolA*, 53Δ*nodD1*, and 53Δ*nodD1nolA* were compared with those of CCBAU53363 on the two hosts. Nodulation of 53Δ*nolA* and 53Δ*nodD1nolA* was delayed about 5 days, while *nodD1* deletion did not affect nodulation kinetics on peanut (Fig. 3A). Meanwhile, *nolA* mutation did not affect the start time of nodulation on mung bean (Fig. 3B). Since 53Δ*nodD1* and 53Δ*nodD1nolA* completely were unable to nodulate mung bean, we included only 53Δ*nodD1* as a representative for nodulation kinetics with no nodule formation in the whole observation period (Fig. 3B). Additionally, the nodulation delay phenotype of 53Δ*nolA* on peanut could be restored to the wild-type level in homologous complementation of *nolA* (Fig. 3A), and *nodD1* from CCBAU53363 or CCBAU51778 could also recover the nodulation speed of 53Δ*nodD1* on mung bean (Fig. 3B). Considering that 53Δ*nolA* and 53Δ*nodD1nolA* exhibited delayed nodule formation on peanut and induced nitrogen-deficient symptom regarding shoot dry weight and leaf chlorophyll content (Fig. 1B and C and Fig. 3A), we supposed the start time for nitrogen fixation of 53Δ*nolA* and 53Δ*nodD1nolA* was also delayed due to the decreased nodulation speed. This estimation was evidenced by the analyses of nitrogenase activity kinetics, in which the nitrogenase activities normalized by the number of peanut plants were significantly lower in nodules induced by both 53Δ*nolA* and 53Δ*nodD1nolA* than those of CCBAU53363 during the period 15 to 40 days postinoculation (dpi) (Fig. 3C), and the nitrogenase activity was significantly lower for 53Δ*nodD1nolA* than for 53Δ*nolA* during the period 25 to 84 dpi (Fig. 3C), which is in line with the aforementioned notion that *nodD1* and *nolA* could make cumulative contributions to CCBAU53363-peanut symbiosis.

In terms of competitive nodule colonization, the nodule occupation ratio of 53Δ*nodD1* and 53Δ*nolA* mutants was significantly lower than that of CCBAU53363, and 53Δ*nodD1nolA* was more severely disadvantaged when competed against CCBAU53363, accounting for only 1.85% of peanut nodules (Fig. 3D). Similarly, 53Δ*nolA* was also significantly outcompeted by CCBAU53363 for nodule colonization on mung bean regardless of the increased nodule formation by 53Δ*nolA* (Fig. 2A and Fig. 3D). Therefore, *nolA* and *nodD1* contribute to the overall competitiveness of CCBAU53363.

### Potential mechanism for the symbiotic defect of 53Δ*nolA* and 53Δ*nodD1nolA* on peanut revealed by transcriptomic analyses.

**FIG 4:**
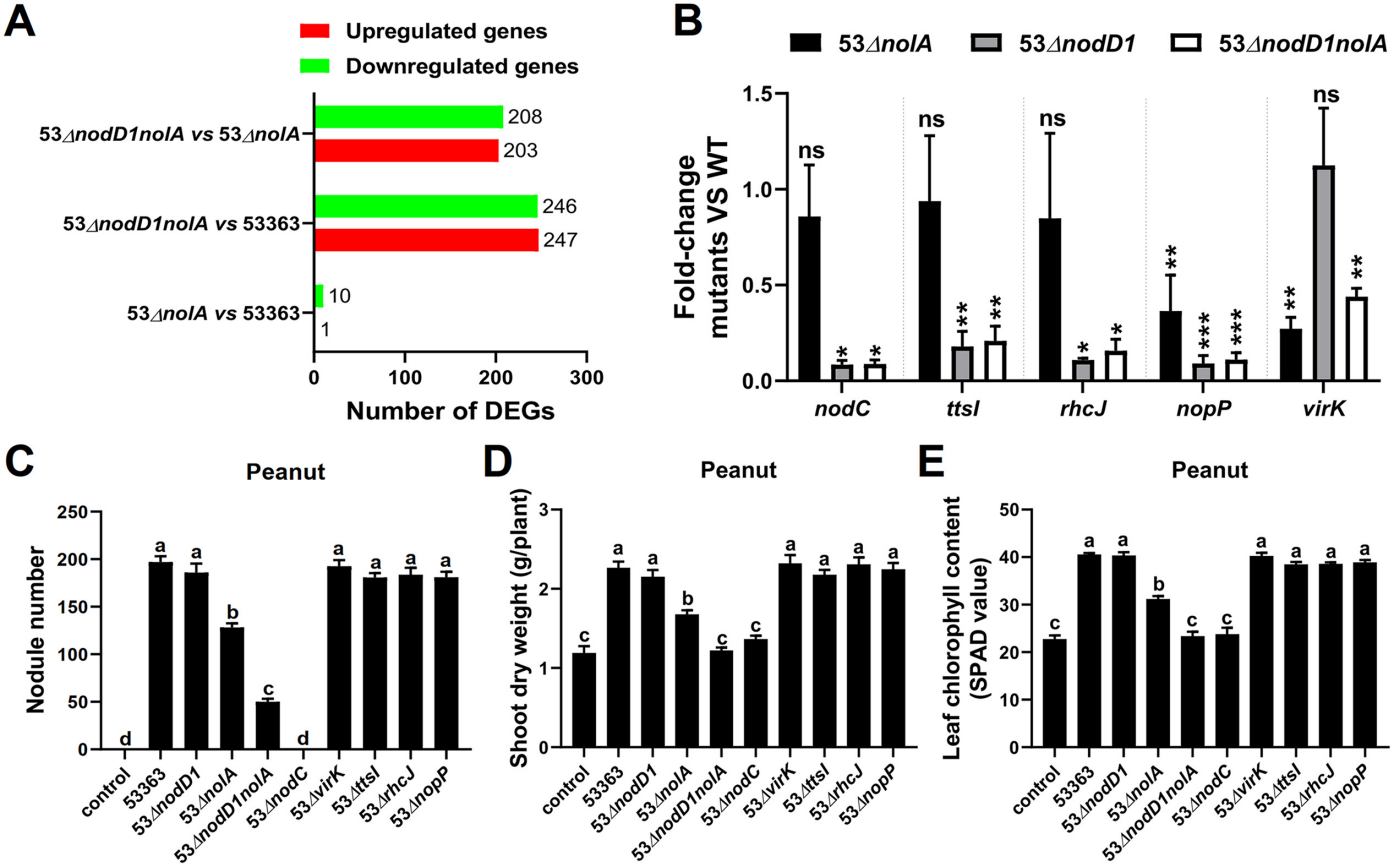
Identification of representative genes putatively regulated by NolA or/and NodD1 via RNA-seq, qRT-PCR, and reverse-genetics analyses on peanut. (A) Transcriptomic profiles of wild-type strain CCBAU53363 and derivate mutants (53Δ*nolA* and 53Δ*nodD1nolA*), which were induced for 22 h in the presence of symbiotic signal genistein (1 μM). The number of significantly down- and upregulated genes is shown for 53Δ*nolA* compared with CCBAU53363, 53Δ*nodD1nolA* compared with CCBAU53363 and 53Δ*nodD1nolA* compared with 53Δ*nolA*. (B) qRT-PCR analysis of *nodC*, *ttsI*, *rhcJ*, *nopP*, and *virK* transcription in wild-type strain CCBAU53363 and derivate mutants (53Δ*nolA*, 53Δ*nodD1*, and 53Δ*nodD1nolA*) induced by 1 μM genistein for 22 h. Results by means ± SEM from nine biological replicates in three independent experiments are shown (*t* test; ns, not significant, *P* > 0.05; *, *P* < 0.05; **, *P* < 0.01; ***, *P* < 0.001). (C to E) Reverse-genetics determination of representative genes in CCBAU53363 interacting with peanut. Shown are the number of infected nodules (C), shoot dry weight (D), and leaf chlorophyll content (E) of peanut inoculated with wild-type strain CCBAU53363 and its derivate strains with mutation of representative genes. Values shown are means ± SEM of values from more than 17 plants scored from three independent experiments (5 to 7 replicate plants per treatment). Different lowercase letters indicate significant differences among means based on Duncan’s test (α = 0.05).

To clarify the reason for the symbiotic defect of 53Δ*nolA* on peanut, transcriptomic analyses of 53Δ*nolA* and CCBAU53363 under induction by genistein were conducted. Only 11 differentially expressed genes (DEGs) were identified (Fig. 4A), including 1 upregulated and 10 downregulated genes in 53Δ*nolA* compared to CCBAU53363 (log_2_ |*R*| > 1; false-discovery rate [FDR] < 0.01). Of the 11 DEGs (Table S2A and B), *nopP*, encoding a T3SS effector, and *virK*, encoding a type IV secretion system (T4SS) protein, were most related to mediating symbiotic compatibility. Further qRT-PCR experiments confirmed that NolA indeed activated the expression of *nopP* and *virK* (Fig. 4B), which is consistent with the transcriptome sequencing (RNA-seq) results. To further investigate whether the decreased expression of *nopP* or *virk* accounted for the symbiotic defect of 53Δ*nolA*, 53Δ*nopP* and 53Δ*virK* were constructed and inoculated on peanut. However, the symbiotic performances of 53Δ*nopP* and 53Δ*virK* were similar with CCBAU53363 (Fig. 4C to E). Then we further deleted another two DEGs regulated by NolA, including *X268_RS37710*, encoding an LLM class flavin-dependent oxidoreductase, and *X268_RS35990*, encoding a transposase, and again no significant difference was observed (Fig. S4A to C), implying the symbiotic defect of 53Δ*nolA* is more likely caused by a synergistic function of multiple DEGs, but we also could not rule out the possibility that the remained uncharacterized DEGs may have impacts on symbiosis of CCBAU53363 on peanut.

**FIG 5:**
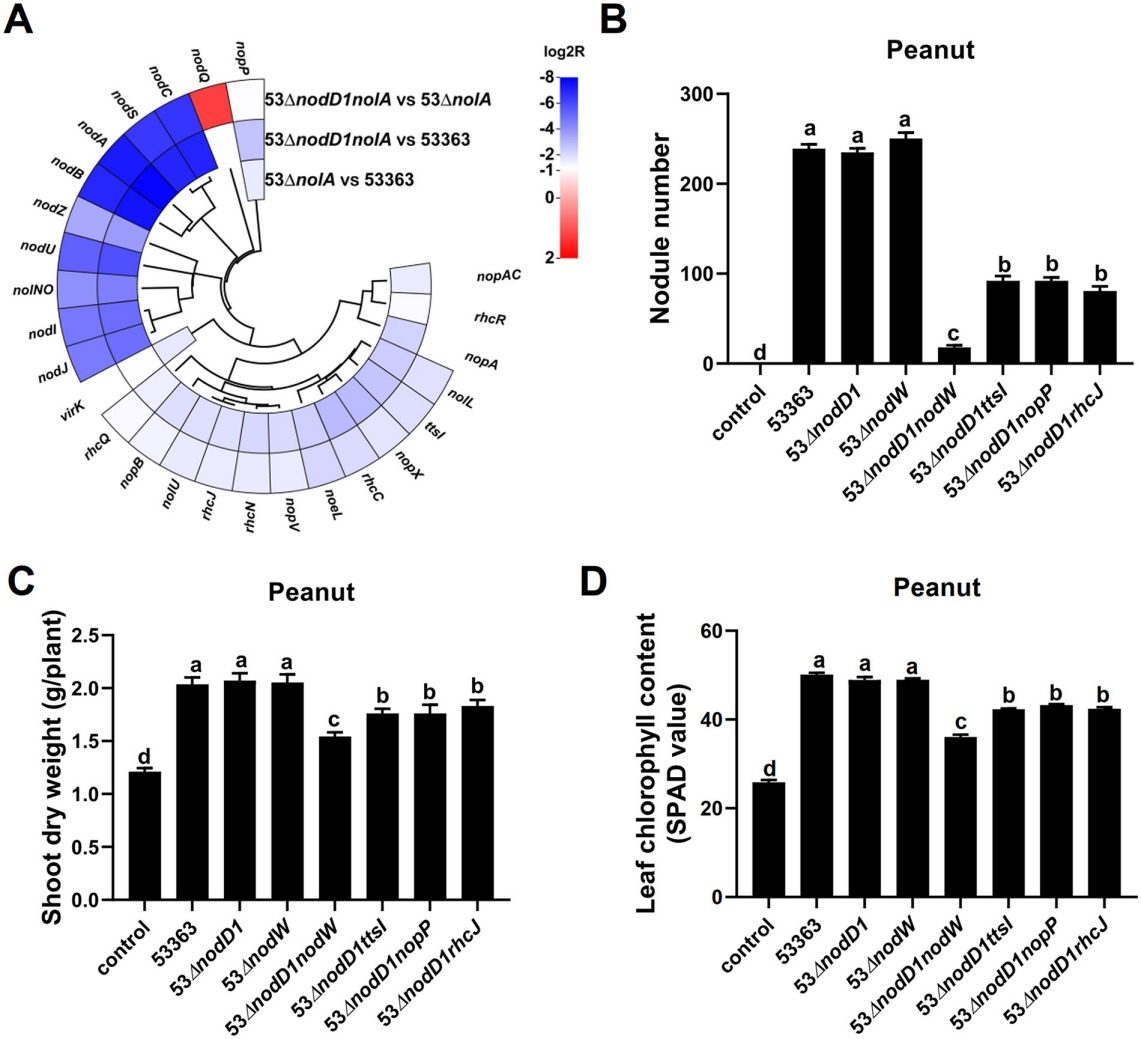
RNA-seq and reverse-genetics analyses revealed some *nod* and T3SS genes differentially expressed in the reciprocal pairs of strains which might account for the symbiotic defect of 53Δ*nodD1nolA*. (A) RNA-seq analysis showed some nodulation and T3SS-related DEGs for 53Δ*nolA* compared with CCBAU53363, 53Δ*nodD1nolA* compared with CCBAU53363, and 53Δ*nodD1nolA* compared with 53Δ*nolA*. Different colors indicate the relative expression levels (log_2_ |*R*| > 1; FDR < 0.01). (B to D) Deletion of representative DEGs in 53Δ*nodD1* to elucidate the symbiotic defect of 53Δ*nodD1nolA* on peanut. Shown are the number of infected nodules (B), shoot dry weight (C), and leaf chlorophyll content (D) of peanut inoculated with wild-type strain CCBAU53363 and its derivate strains with mutation of representative genes. Values shown are means ± SEM from more than 20 plants scored from three independent experiments (6 to 8 replicate plants per treatment). Different lowercase letters indicate significant differences among means based on Duncan’s test (α = 0.05).

Considering more abnormal bumps were induced by 53Δ*nodD1nolA* than by 53Δ*nolA* (Fig. S3A), RNA-seq analysis revealed that 493 DEGs between 53Δ*nodD1nolA* and CCBAU53363 and 411 DEGs between 53Δ*nodD1nolA* and 53Δ*nolA* were identified (Fig. 4A). The expression profiles of the symbiosis-related genes, containing those related to nodulation, T3SS/T4SS/T6SS, and surface polysaccharides (37, 38) are shown in Table S2C. Notably, most of the nodulation- and T3SS-related genes were significantly downregulated in 53Δ*nodD1nolA* compared to those in CCBAU53363 (Fig. 5A and Table S2C). Meanwhile, *nodC*, *ttsI*, *rhcJ*, and *nopP* were also further downregulated in 53Δ*nodD1nolA* compared to those in 53Δ*nolA* (Fig. 5A and Table S2C), indicating that NodD1 positively regulated *nod*/T3SS-related genes, at least in the *nolA* deletion background. qRT-PCR experiments confirmed that NodD1 was an important transcriptional activator of *nodC*, *ttsI*, *rhcJ*, and *nopP* (Fig. 4B), and TtsI also activated the transcription of *rhcJ* and *nopP* (Fig. S4D), while no mutually regulated relationship between *nolA* and *nodD1* was identified (Fig. S4E). Although *nopP* is the only significantly downregulated gene in either 53Δ*nolA* versus CCBAU53363, 53Δ*nodD1nolA* versus CCBAU53363 or even 53Δ*nodD1nolA* versus 53Δ*nolA* comparison group (Fig. 5A and Table S2C), *nopP* deletion did not exert symbiotic defect on peanut as mentioned above (Fig. 4C to E). Also, the corresponding mutants of DEGs, including 53Δ*nodC*, 53Δ*ttsI*, and 53Δ*rhcJ*, showed no significant defect on peanut except 53Δ*nodC*, which was completely unable to nodulate peanut (Fig. 4C to E), demonstrating that NFs are essential for CCBAU53363 symbiosis with peanut.

Moreover, *nodD1* deletion did not affect CCBAU53363 symbiosis with peanut but rendered decreased expression of *nod*/T3SS-related genes (Fig. 1 and Fig. 4B), indicating that the downregulated levels of *nod*/T3SS-related genes might not be sufficient to deprive 53Δ*nodD1* of the normal symbiotic ability, possibly due to the presence of some other regulators, like NodW in USDA110, which provided an alternative pathway to activate *nod*/T3SS-related genes in the absence of NodD1 (16, 39). In line with this hypothesis, NodW indeed activated the expression of *nodC*, *nodD1* and *ttsI* in CCBAU53363 (Fig. S4F). Additionally, compared to the symbiotic performances of 53Δ*nodD1* and 53Δ*nodW*, which were similar to that of CCBAU53363, the symbiotic ability of 53Δ*nodD1nodW* was significantly impaired (Fig. 5B to D). Since *nodD1nolA* double mutation rendered more severe symbiotic defect on peanut than *nolA* mutation, and 53Δ*nodD1* could efficiently nodulate peanut (Fig. 1 and Fig. S3A), indicating *nodD1* exerted some effects on symbiotic adaptation in the *nolA* deletion background. *nopP* rather than *nodC*, *ttsI*, and *rhcJ* was positively regulated by NolA, while NodD1 activated all four genes (Fig. 4B); hence we supposed that the further downregulation of *nopP* in 53Δ*nodD1nolA* compared to 53Δ*nodD1* might be highly possible to explain the symbiotic defect of 53Δ*nodD1nolA* on peanut. To verify this, we deleted *nopP* in the *nodD1* deletion background and inoculated 53Δ*nodD1nopP* on peanut. Significant symbiotic defects of 53Δ*nodD1nopP* on peanut were observed compared to those of 53Δ*nodD1* or CCBAU53363 (Fig. 5B to D). We further deleted another two T3SS-related genes in the *nodD1* deletion background and symbiotic performances of 53Δ*nodD1 ttsI* and 53Δ*nodD1rhcJ* on peanut also presented decreased nodule numbers and plant growth traits (Fig. 5B to D), implying that T3SS-related genes are important for symbiosis of 53Δ*nodD1* with peanut, and the further downregulation of *nopP* in 53Δ*nodD1* whose *nod*/T3SS-related genes were downregulated might render symbiotic defect on peanut just like 53Δ*nodD1nolA* did. However, similar transcriptional levels of *nopP* in 53Δ*nodD1* and 53Δ*nodD1nolA* were detected by qRT-PCR (Fig. 4B), Therefore, *nodD1* did not cumulatively contribute with *nolA* to the transcriptional levels of *nopP*, implying that NolA might activate *nopP* expression at the posttranscriptional level or other unknown DEGs coordinately regulated by NolA and NodD1 might account for the symbiotic defect of 53Δ*nodD1nolA* on peanut. Furthermore, an electrophoretic mobility shift assay (EMSA) was performed to test whether NolA could directly regulate *nopP*. When the concentration of purified glutathione *S*-transferase (GST)-NolA protein was increased, the *nopP* promoter probe showed no gel shift pattern of NolA binding (Fig. S5D), suggesting an indirect regulation of *nopP* by NolA. Collectively, CCBAU53363-peanut symbiosis is NF dependent, which could be mediated by synergistic function of nodulation/T3SS-related genes under sophisticated regulation of NodD1 and NolA, and T3SS-related genes could optimize this symbiotic process under a decreased-NF condition derived from *nodD1* deletion.

### Repressed nodulation of CCBAU53363 on mung bean by NolA via *nopP* activation.

**FIG 6:**
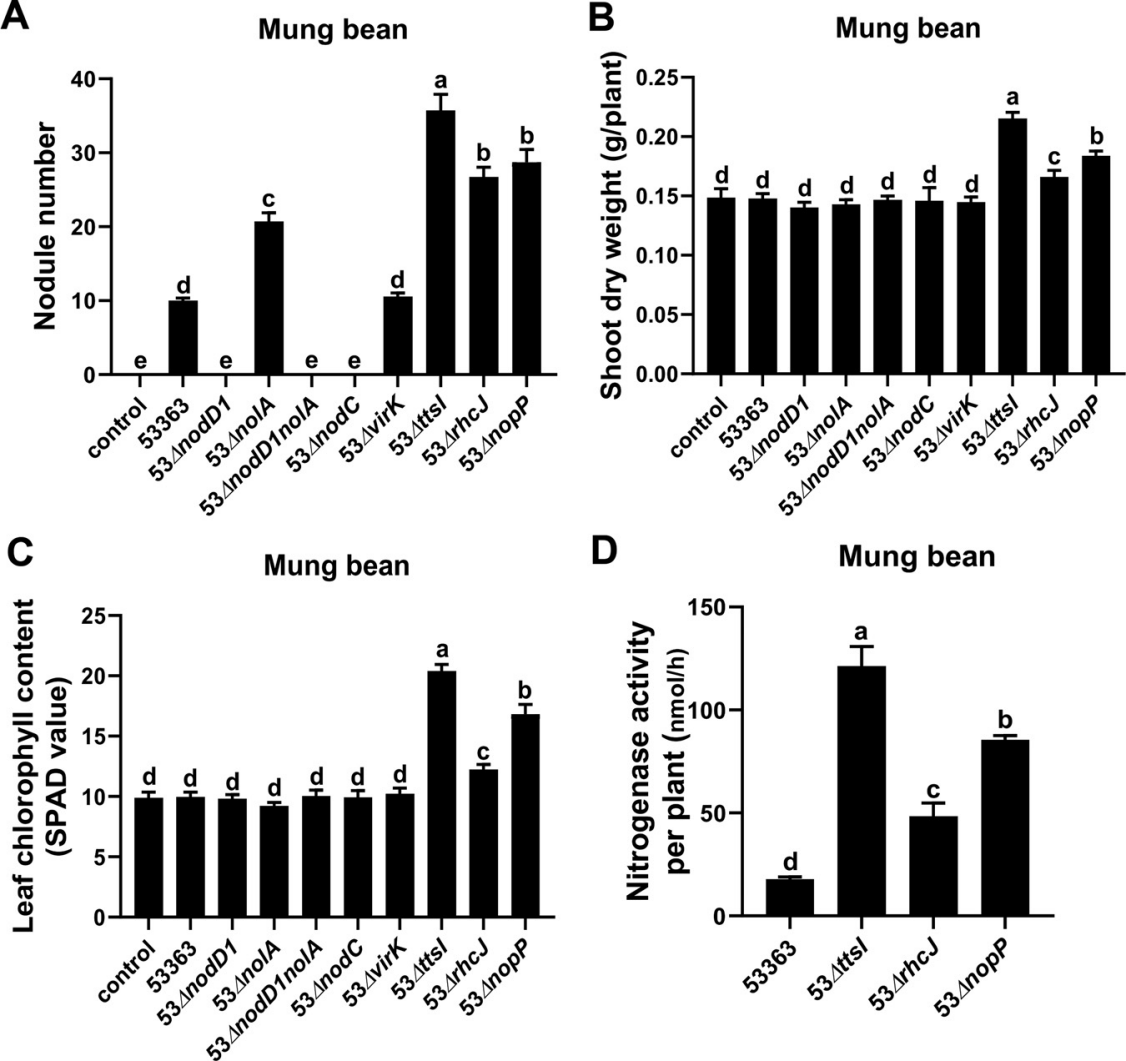
New “repressors” were found to restrict CCBAU53363 to nodulate mung bean. (A to D) Deletion of representative T3SS genes in CCBAU53363 to enhance the symbiotic capability on mung bean. Shown are the number of infected nodules (A), shoot dry weight (B), leaf chlorophyll content (C), and nitrogenase activity levels per plant (D) of mung bean inoculated with wild-type strain CCBAU53363 and its derivate strains with mutation of representative genes. Values shown are means ± SEM of values from more than 15 plants scored from three independent experiments (5 or 6 replicate plants per treatment) at 30 dpi. Different lowercase letters indicate significant differences among means based on Duncan’s test (α = 0.05).

To unveil potential symbiosis “players” regulated by NolA that caused the repressive effect on nodulation of CCBAU53363 with mung bean, 53Δ*nopP*, 53Δ*virK*, 53Δ*X268_RS37710*, and 53Δ*X268_RS35990* were individually inoculated on mung bean. Only 53Δ*nopP* induced a significantly increased number of nodules compared with CCBAU53363 (Fig. 6A and Fig. S5A). More importantly, the shoot dry weight and leaf chlorophyll content also significantly increased (Fig. 6B and C and Fig. S5B and C), indicating that 53Δ*nopP* could more effectively nodulate mung bean than CCBAU53363 or 53Δ*nolA*. Indeed, the nitrogenase activity per plant was also significantly higher in nodules induced by 53Δ*nopP* than in those induced by CCBAU53363 (Fig. 6D). The repressive role of *nopP* raised an intriguing hypothesis that T3SS-related genes might act as important repressors accounting for ineffective symbiosis between CCBAU53363 and mung bean. In line with this hypothesis, inoculation with 53Δ*ttsI* and 53Δ*rhcJ* mutants also significantly increased symbiotic performances compared to CCBAU53363 on mung bean (Fig. 6A to C). Additionally, the nitrogenase activities per plant differed significantly among plants inoculated with 53Δ*ttsI*, 53Δ*nopP*, 53Δ*rhcJ*, and CCBAU53363, with the greatest nitrogenase activity in the 53Δ*ttsI*-inoculated plants, followed in order by 53Δ*nopP*, 53Δ*rhcJ*, and CCBAU53363 (Fig. 6D), and this ordinal pattern is consistent with those in their corresponding shoot dry weight and leaf chlorophyll content (Fig. 6B and C). Moreover, neither RNA-seq analysis of NolA regulon nor qRT-PCR experiments showed the regulation of *ttsI* and *rhcJ* by NolA (Fig. 4B and Table S2C). These results indicated that other T3SS-related genes besides *nopP* inhibit the effective symbiosis between CCBAU53363 and mung bean, but only *nopP* is positively regulated by NolA. NolA was previously reported to repress *nod* expression (13, 18), but our results did not identify this regulatory mode (Fig. 4B and Table S2C). Similar to peanut, mung bean could not form nodules with 53Δ*nodC* (Fig. 6A), which also means a NF-dependent way for CCBAU53363 to nodulate mung bean.

## DISCUSSION

The establishment of symbiosis between bradyrhizobia and their hosts generally involves “cross talk” regulation between members of three global regulatory families, including NodD, NolA, and NodW, which controlled the *nod* gene expression for starting the early nodulation events (13, 40). In this study, we evidenced that the functions of *nodD1* and *nolA* presented a host-dependent feature in CCBAU53363. Our findings also added new insight for the regulation function of *nodD1*. Although different NodD proteins activate or repress *nod* gene transcription in the presence of appropriate inducers and their regulations are complicated (11, 12, 33, 41), at least one copy of *nodD* is essential for nodulation in rhizobial species (9, 10). However, *nodD1* was dispensable for CCBAU53363 nodulation of peanut but essential for nodulation of mung bean, suggesting a host-dependent manner (Fig. 1, Fig. 2, Fig. 4C to E, and Fig. 6A to C). Due to the dispensable role of *nodD1* in CCBAU53363-peanut symbiosis, we supposed NodW provided an alternative way for activation of *nod* genes, just like NodW in USDA110 (16). This estimation was supported by the results that 53Δ*nodW* and 53Δ*nodD1* did not affect their efficient symbiosis with peanut, but 53Δ*nodD1nodW* significantly decreased the symbiotic efficiency (Fig. 5B to D). So, both NodD1 and NodW could independently activate *nod* gene expression in CCBAU53363 symbiosis with peanut, and we also demonstrated NodW activated *nodD1* and *ttsI* besides *nodC* (see Fig. S4F in the supplemental material). However, NodW was more likely a repressor for CCBAU53363 nodulating mung bean, since 53Δ*nodW* significantly enhanced the symbiotic ability with mung bean (Fig. S6), demonstrating a novel function for NodW in regulating symbiotic compatibility. Therefore, *nodD1* and *nodW* might cooperatively function in different ways during the symbiotic processes of CCBAU53363 with different hosts. On the other hand, NolA indirectly inhibited *nodABC* expression by promoting *nodD2* expression in USDA110 (15, 19), while NolA and NodD1 did not regulate *nodA* under induction by genistein in strain NC92 (27). However, NolA of CCBAU53363 did not regulate the expression of *nodC*, which was activated by NodD1 nevertheless (Fig. 4B).

Possible regulatory relationship was also revealed by transcriptomic analyses for explaining the discrepancy in symbiotic performance of CCBUAU53363 with the two hosts, and the significant downregulation of *nopP* and *virK* in 53Δ*nolA* compared to CCBAU53363 (Table S2), and further decreases in *nopP* expression in 53Δ*nodD1nolA* compared to 53Δ*nolA* (Fig. 5A and Table S2C) were found. Indeed, T3SS/T4SS-related genes were reported to have different effects on symbiosis which is host dependent (42, 43). For instance, compared with the corresponding wild-type strains, *B. elkanii* SEMIA587 Δ*ttsI* formed fewer nodules on cowpea and showed no symbiotic defects on siratro (44), but *S. fredii* NGR234 Δ*ttsI* had increased nodule number on jicama (Pachyrhizus tuberosus) (45). *B. elkanii* USDA61 Δ*rhcJ* failed to nodulate on Indian jointvetch (Aeschynomene indica) (46), and symbiosis of *S*. *fredii* HH103 Δ*rhcJ* with soybean was impaired (47). *nopP* mutation of *S. fredii* HH103 (48) or NGR234 (49, 50) also had different effects on symbiosis with different legumes; these host-dependent effects were possibly due to effector-triggered immunity mediated by different R proteins in different legumes. Here, *virK* deletion had no impact on CCBAU53363 symbioses with peanut and mung bean (Fig. 4C to E and Fig. 6A to C), although the *vir* cluster mutants in Mesorhizobium loti R7A (51) and Sinorhizobium meliloti KH46c (52) exhibited host-dependent symbiotic interaction on different legume species. However, 53 Δ*nopP* exhibited a higher symbiotic capacity than CCBAU53363 on mung bean instead of peanut (Fig. 4C to E and Fig. 6). Therefore, the enhanced symbiotic capability of 53Δ*nolA* might be due to the downregulated expression of *nopP*, and the symbiotic defect of 53Δ*nolA* or 53Δ*nodD1nolA* on peanut might be caused by differential expression of multiple accessory genes besides *nopP* and *virK*. Indeed, two DEG sets in 53Δ*nolA* and 53Δ*nodD1nolA* compared to CCBAU53363 were, respectively, obtained by transcriptomic analyses, which showed 11 shared DEGs and 482 DEGs unique to 53Δ*nodD1nolA* (Fig. S7A), and 1 of the 11 common DEGs was significantly differentially expressed in 53Δ*nodD1nolA* compared to 53Δ*nolA* (Fig. S7B). *nolA* exerted a cumulative effect with *nodD1* on symbiotic performance on peanut, with more poorly infected root bumps, and fewer infected nodules induced by 53Δ*nodD1nolA* than 53Δ*nolA* were observed (Fig. 1 and Fig. S3A), implying that the formation of poorly infected root bumps and reduced nodule number may be mediated by effector-triggered immunity (53). To further reveal the reason for the weakened symbiotic ability of 53Δ*nodD1nolA*, the above 482 unique DEGs in 53Δ*nodD1nolA* and 1 common DEG were combined as a new gene set. Gene Ontology (GO) enrichment analysis of the 483 DEGs showed that 254 (52.6%) had GO annotation information, and they were significantly enriched in 76 GO terms (FDR < 0.05), of which 24, 14, and 38 GO terms, respectively, belonged to the biological process, cellular component, and molecular function categories, respectively (Table S3). The top 15 extremely enriched GO terms (FDR < 0.01), displayed in a bubble diagram in Fig. S7C, belonged to biological process categories such as symbiotic process (GO:0044403) and nodulation (GO:0009877), a cellular component category such as transmembrane transporter complex (GO:1902495), and molecular function categories such as hydrogenase (acceptor) activity (GO:0033748) and ATPase activity (GO:0016887). The expression of nodulation/T3SS-related DEGs in GO terms (GO:0044403 and GO:0009877) was significantly downregulated in 53Δ*nodD1nolA*. Other DEGs besides the nodulation/T3SS-related DEGs might also play important roles and account for the symbiotic defect of 53Δ*nodD1nolA* on peanut.

Several “repressors” were previously screened to account for the symbiotic incompatibility between CCBAU53363 and mung bean (29). Herein, we showed that *nolA*, *nodW*, as well as T3SS-related genes, including *nopP*, *ttsI*, and *rhcJ* served as new “repressors” for inhibiting efficient symbiosis of CCBAU53363 with mung bean (Fig. 6 and Fig. S6). Moreover, we also demonstrated NolA repressed nodulation of CCBAU53363 on mung bean via *nopP* activation. In *B. diazoefficiens* USDA122, three critical amino acid residues (R60, R67, and H173) of NopP sequence determined the bradyrhizobial incompatibility with *Rj2* of soybean, resulting in activation of the soybean-defense marker gene *PR-2* (54). Due to *Rj2* orthologs conserved in mung bean, inoculation with USDA110 carrying *nopP*_122_ also resulted in symbiotic defect on mung bean than inoculation with USDA110 having internal *nopP*_110_, while *nopP*_110_ could enable USDA122 to form significantly more nodules and had a higher biomass on mung bean (54). It should be noted that the NopP of CCBAU53363 also retains two of the three critical amino acid residues in NopP_122_ (Fig. S8): whether these two amino acid sites contributed to the symbiotic incompatibility of CCBAU53363 with mung bean should be determined in future study.

The symbiosis between rhizobia and legumes is influenced by many molecular signals, including NFs and secreted proteins, and T3SS could bypass the NFs’ recognition for nodule formation in some bradyrhizobia (55), while *nopP* inactivation enhanced the symbiotic ability of CCBAU53363 on mung bean (Fig. 6), and this interaction was strictly NF dependent (Fig. 6A). In *Bradyrhizobium* sp. strain ORS285, the role of T3SS was dependent on diverse NF-dependent and NF-independent *Aeschynomene* plants, indicating a mutual but complicate relationship between *nod* genes and T3SS genes (46). *Arachis* is phylogenetically close to *Aeschynomene* (25, 56), and like *Aeschynomene* plants, peanut could also support *nod*-dependent or *nod*-independent symbiosis with different bradyrhizobia (8, 23, 26). 53Δ*nodC* did not nodulate peanut (Fig. 4C), demonstrating that the symbiosis between CCBAU53363 and peanut is NF dependent, although 53Δ*nodD1* established efficient symbiosis, putatively due to the fact that *nod* genes could be activated by other alternative regulators such as NodW or low expression of *nod* genes is sufficient for symbiosis. Additionally, *nodD1* exerted cumulative effects with T3SS-related genes on symbiotic capability on peanut (Fig. 4C to E and Fig. 5B to D). These findings in combination with the results that *nodC* is required for symbiosis and monogenic mutation of T3SS-related genes did not affect symbiosis inspired us to conclude that nodulation genes predominantly function jointly with T3SS genes, contributing to efficient symbiosis on peanut. Therefore, the transcription of key symbiotic genes involved in the early symbiotic interaction modulated by NodD1 and/or NolA is crucial for optimizing symbiotic compatibility, although the detailed mechanisms underlying this link remain elusive.

### Conclusions.

**FIG 7:**
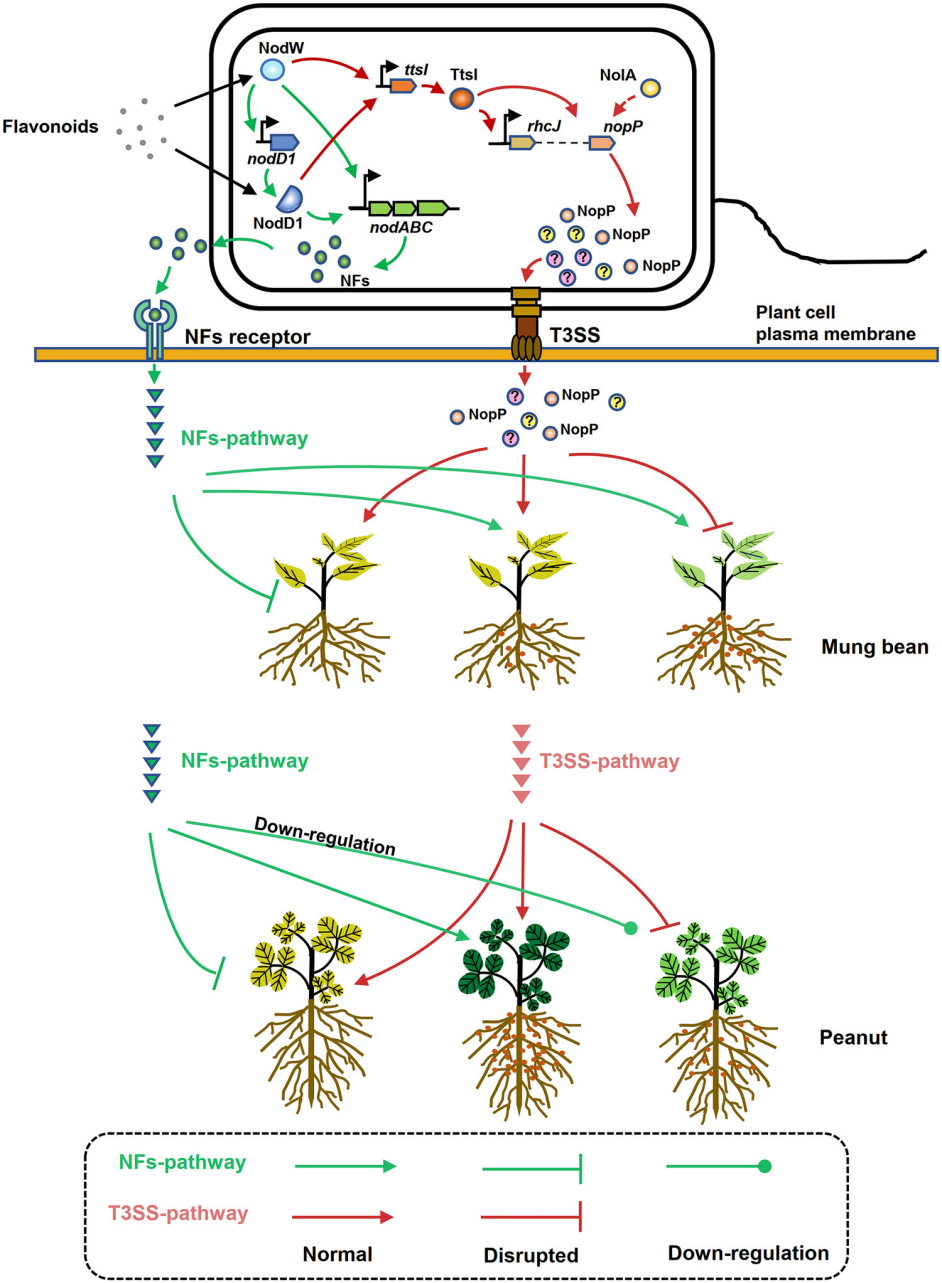
Proposed working model for the role of NFs and T3SS components regulated by NolA and/or NodD1 in modulating symbiotic compatibility. The NF pathway and T3SS pathway are shown in green and red, respectively. In CCBAU53363, NodD1 activated the transcription of *nodC* and *ttsI*, while TtsI activated *rhcJ* and *nopP*. NolA also positively regulated *nopP* expression. T3SS components restricted nodulation between CCBAU53363 and mung bean. When the T3SS pathway is disrupted, the symbiotic capability of CCBAU53363 on mung bean was improved. When the NF pathway is disrupted, CCBAU53363 could not establish symbioses with mung bean and peanut. The integrity of the NF pathway predominantly functions to ensure the normal symbiosis between CCBAU53363 and peanut, but the downregulation level to some extent of *nod* genes joined with disruption of T3SS-related genes also rendered the symbiotic defect of CCBAU53363 on peanut.

In this study, we revealed the important roles of NFs and T3SS components that were regulated by NodD1 and/or NolA in modulating symbiotic adaptations of CCBAU53363 with peanut and mung bean. NodD1 activated the transcription of *nodC* and *ttsI*, which in turn activated *rhcJ* and *nopP* (Fig. 7). NolA only positively regulated expression of *nopP* instead of *nodC*, *rhcJ*, and *ttsI*, and NodW activated *nodD1*, *nodC*, and *ttsI* (Fig. 7). Therefore, NodD1 is involved in both NF and T3SS pathway regulation cascades, while NolA is only related to the T3SS pathway mediated by NopP. The NF pathway is essential for CCBAU53363 nodulation of both hosts. The T3SS pathway restricts the nodulation capability of CCBAU53363 on mung bean. However, the T3SS pathway sustains efficient symbiosis of CCBAU53363 on peanut when *nodD1* is disrupted. That is, downregulation of NF-related genes combined with the disruption of T3SS-pathway results in symbiotic defect of CCBAU53363 on peanut (Fig. 7). To this end, this study advances our understanding of the regulatory roles of NolA and NodD in the symbiotic adaptations of bradyrhizobia with different hosts.

## MATERIALS AND METHODS

### Bacterial strains, plasmids, primers and growth conditions.

All bacterial strains and plasmids used in this study are listed in Table S4A in the supplemental material. *B. guangxiense* CCBAU53363 and its derivates were cultured at 28°C in tryptone-yeast extract (TY) medium, yeast-mannitol agar (YMA) medium, or yeast extract-mannitol (YEM) medium (57, 58). All Escherichia coli strains were cultured in Luria-Bertani (LB) medium at 37°C (59). Antibiotics were added at concentrations described previously when necessary (29). The TY medium was supplemented with 5% (wt/vol) sucrose when required.

### Construction and complementation of CCBAU53363 mutants.

All primers and their specific roles in this study are listed and described in Table S4B. The suicide plasmid pJQ200SK (60) was used to delete one or more genes. Briefly, a pJQ200SK derivative harboring two PCR fragments encompassing the upstream and the downstream regions of the target gene was constructed and subsequently conjugated into the recipient strain with the helper plasmid pRK2013 (61). Details of the seamless cloning approach, triparental conjugation procedures, and methods for screening single- or double-crossover transconjugants are given in previous studies (29, 53, 62). In detailed, the seamless cloning approach is described as follows. The 5′-flanking and 3′-flanking fragments (700 to 1,000 bp) of the target gene were obtained by PCR amplification using 2×High-GC PCR StarMix (GenStar), and the two resulting PCR fragments were then cloned into the linearized SmaI-digested pJQ200SK using Master assembly mix (Clone Smarter) at 50°C for 15 min to obtain the pJQ200SK-derived plasmids. The triparental conjugation was then carried out using recipient *B. guangxiense* CCBAU53363 or its derivate mutants, the donor bacteria harboring pJQ200SK-derived plasmid, and the auxiliary bacteria containing the helper plasmid pRK2013 by washing twice with TY liquid medium. Then, recipient bacteria, donor bacteria and auxiliary bacteria were, respectively, sampled in a ratio of 100:7:3 and mixed gently. The final bacterial mixture was added dropwise to TY agar plates without any antibiotics. After culture at 28°C chamber for 4 days, screening of single- or double-crossover transconjugants was performed; single-crossover transconjugants were screened for resistance to gentamicin, followed by cell passage cultivation and counterselection for double recombinants on TY agar plates containing 5% (wt/vol) sucrose. Polygenic mutants were generated by transforming pJQ200SK derivatives into the appropriate monogenic mutants. pJQ200SK derivatives were also used to transfer homologous or heterogenous genes into the mutants *in situ*, which not only contained the upstream and the downstream regions of the genomic region to be complemented but also contained the homologous or heterogenous target genes. All of the plasmids and derivatives of CCBAU53363 were verified using clone PCR and Sanger sequencing.

### Plant assays, cytological observations, and competitive nodule colonization assays.

Seeds of peanut and mung bean were surface sterilized in 25% (vol/vol) or 20% (vol/vol) NaClO for 15 min and 10 min, respectively. The sterilized seeds were rinsed with sterile distilled water 7 to 10 times and then transferred onto 0.6% (wt/vol) agar plates for germination at 28°C in darkness. Seedlings were inoculated with 1 mL of the appropriate bacterial culture once the culture reached an optical density at 600 nm (OD_600_) of 0.25. Inoculated seedlings were grown in vermiculite moistened with low-N nutrient solution [0.03 g/L Ca(NO_3_)_2_·4H_2_O, 0.075 g/L KCl, 0.06 g/L MgSO_4_·7H_2_O, 0.136 g/L K_2_HPO_4_, 0.46 g/L CaSO_4_·2H_2_O, 0.075 g/L ferric citrate, 10 mL trace element mixture (2.86 g/L H_3_BO_3_, 1.81 g/L MnSO_4_, 0.8 g/L CuSO_4_·5H_2_O, 0.22 g/L ZnSO_4_, 0.02 g/L H_2_MoO_4_)] at 24°C under a day/night cycle of 12 h/12 h. At 45 days postinoculation (dpi) (peanut) or 30 dpi (mung bean), nodule number, shoot dry weight, and leaf chlorophyll content were determined as described previously (63). Leaf chlorophyll content was measured using a SPAD-502 chlorophyll meter in three leaflets from the third leaf (peanut) or the first leaf (mung bean). Meanwhile, nodules of peanut or mung bean were harvested and fixed using 2.5% (vol/vol) glutaraldehyde in 0.05 M cacodylate buffer for cytological observation. Ultrathin sections of the fixed nodules were prepared as described previously (64) and observed under a JEM-1230 transmission electron microscope (TEM).

For the competitive nodule colonization assay, equal volumes of bacterial culture (OD_600_ equivalent to 0.25) were mixed and seedlings were inoculated with 1 mL of this culture. Nodules from three to five plants were collected at 45 dpi (peanut) or 30 dpi (mung bean) and surface sterilized by shaking for 30 s in 95% ethanol, followed by 5 min or 3 min in a 20% (vol/vol) NaClO solution, respectively. Then the sterilized nodules were crushed after washing seven times with sterile water, and the exudate was streaked onto a YMA plate. Following 7 to 10 days of growth at 28°C, the wild type and mutants were verified by clone PCR and Sanger sequencing.

### Measurement of nitrogenase activity and nodulation kinetics assays.

To explain the causes for nitrogen deficiency phenotypes of the host plants, nitrogenase activity was measured using the acetylene reduction method as previously described (65). Nitrogenase activity per plant was used to measure total nitrogen fixation content. Specifically, the peanut or mung bean plant roots were washed to remove vermiculite and dry up the water with absorbent paper. Then the whole roots were separately put into the reaction flask (being careful during the process to avoid the root nodules falling off the roots), and seal it. Ten milliliters of air was drawn out from the reaction bottle and 10 mL of acetylene gas was injected, followed by a reaction in a 28°C incubator for 2 h. At this moment, the reaction gas storage bottles were prepared by withdrawing 2 mL air from the 10-mL serum bottles, then injecting 1 mL double-distilled H_2_O (ddH_2_O), sealing the bottles, and inverting them for use. After 2 h, 1 mL reaction gas was injected into the storage bottles, which were inverted. A gas chromatograph (Agilent 8890) was used to measure the peak area of ethylene gas in 100 μL reaction gas, and the mole numbers of ethylene based on the standard peak area of ethylene were calculated to finally obtain nitrogenase activity per plant.

For nodulation kinetics assays, the number of nodules on the roots of the peanut plants were counted for the first time at 10 dpi and each subsequent day until 17 dpi and then at 20, 25, 30, 35, 45, 55, 72, and 84 dpi. In addition, the numbers of nodules on mung bean roots were counted every day from 7 dpi to 10 dpi and then at 15, 20, 25, 31, 36, 43, 49, 60, and 70 dpi. Three to five plants (peanut or mung bean) per treatment were harvested at each time point.

### RNA isolation, bioinformatic procedures, and qRT-PCR assay.

Wild-type CCBAU53363 and mutants were precultured in 50-mL aliquots of YEM medium at 28°C with shaking at 180 rpm. After 72 h, the precultured strains were transferred to new YEM medium and cultured in triplicate. Early mid-log-phase bradyrhizobial cultures were induced by 1 μM genistein for 22 h. The bradyrhizobial cultures were then harvested at 4°C, and total RNA was extracted as previously described (66). Specifically, 50 mL bradyrhizobial cells were collected by centrifugation and the pellet was ground using a liquid nitrogen precooled mortar. The ground cells were transferred into 1.5-mL RNase-free tubes and homogenized with 1 mL RNAiso Plus reagent (TaKaRa). This new mixture was centrifuged (13,000 × *g* for 20 min at 4°C) after setting at room temperature for 10 min and the superior phase was transferred to another tube. After a wash step with 200 μL chloroform, RNA was precipitated by adding equal volumes of cold isopropanol and high-salt precipitation solution (TaKaRa), mixed with gentle inversion, and let stand for 10 min at room temperature and overnight at −20°C, followed by centrifugation at 13,000 × *g* at 4°C for 10 min, and the supernatant was discarded. After washing twice with 70% ethanol, the resulting RNA was subjected to air drying and dissolved in RNase-free water. The RNA concentrations were detected using a NanoDrop instrument (ND-1000; Thermo Fisher Scientific), and RNA integrity was checked using gel electrophoresis. Further transcriptomic library construction and strand-specific RNA sequencing were performed by Shanghai Majorbio Bio-pharm Technology Co., Ltd. The clean RNA-seq reads were mapped to the CCBAU53363 reference genome using Bowtie2 (67). Unique mapped read numbers for each protein-coding gene were extracted from sorted bam files using HTseq-count (68). Finally, we used DESeq2 to identify differentially expressed genes using the criteria log_2_ |*R*| of >1 and false-discovery rate (FDR) of <0.01 (69). DEGs were functionally annotated against Gene Ontology (GO) (70) using BLAST (71). GO enrichment analysis was performed on the annotated genes, mainly belonging to the biological process, cellular component, and molecular function categories, by GOATOOLS software (72). A neighbor-joining phylogenic tree based on NodD proteins was conducted in MEGA X (73). Their evolutionary distances were computed using the JTT matrix-based method (74). The NopP protein sequence alignment was conducted using MAGE X (73), and the resulting file was further edited by BioEdit (75).

For the qRT-PCR assay, cDNA was synthesized using the FastKing RT kit with gDNase (TianGen), following the manufacturer’s protocols. Using the cDNA as a template, qRT-PCR was performed using 2× RealStar Green Power mixture with ROXII (GenStar) on a QuantStudioT 6 flex system real-time PCR system (ABI). Specifically, the reaction mixture contained 5 μL cDNA, 0.75 μL primer-F, 0.75 μL primer-R, 7.5 μL 2× RealStar Green Power mixture with ROXII, and 1 μL nuclease-free water. Transcription levels of target genes in each sample were normalized against the expression of the internal control gene 16S rRNA in the same sample. The cycling conditions were as follows: 50°C for 2 min and 95°C for 15 min, followed by 40 cycles of 94°C for 20 s and 60°C for 45 s, 95°C for 15 s, 60°C for 1 min, and 95°C for 15 s. Three independent biological experiments were performed for each analysis.

### Purification of NolA and electrophoretic mobility shift assay (EMSA).

The PCR product of NolA amplified using primers 53NolA-F/53NolA-R, SmaI-digested pGEX-KG vector (76) were subject to seamless assembly using Master assembly mix (Clone Smarter) at 50°C for 15 min to generate pGEX-NolA, which was finally transformed into E. coli BL21(DE3). The expression of N-terminal GST-tagged NolA in E. coli cells grown in LB medium was induced with 0.5 mM (final concentration) isopropyl-d-thiogalactopyranoside (IPTG) for 4 h at 37°C. Cells were then harvested, washed, resuspended using lysis buffer (50 mM NaH_2_PO_4_, 300 mM NaCl) with protease inhibitor cocktail, and sonicated on ice. Cell extracts were loaded onto glutathione (GSH)-coupled agarose beads (GenStar), and NolA protein was purified in accordance with the manufacturer’s instructions. The concentration of NolA protein was determined by the Bradford method using Quick Start Bradford dye reagent (Bio-Rad).

Binding reactions were performed with ~12 μM CY5-labeled probes and increased concentrations of purified NolA (0.83,1.67, and 3.33 μM, respectively) in 10 μL of the reaction buffer (1× binding buffer, 1 mg/mL bovine serum albumin [BSA], 1 mg/mL salmon sperm DNA), followed by incubation at 25°C for 30 min in the darkness. The binding mixture was subjected to electrophoresis on 6% (wt/vol) native polyacrylamide gels at 100 V in TB buffer (44.5 mM Tris, 44.5 mM boric acid) for 2 to 3 h in an ice bath. After electrophoresis, fluorescence signals from CY5-labeled DNAs were detected by a Typhoon FLA 9000 imager (GE Healthcare).

### Data availability.

Raw RNA-seq data in this study have been deposited in the National Center for Biotechnology Information (NCBI) Sequence Read Archive server under accession no. PRJNA768971.
