# Supplementary material for: Scrutiny of NolA and NodD1 Regulatory Roles in Symbiotic Compatibility Unveils New Insights into Bradyrhizobium guangxiense CCBAU53363 Interacting with Peanut (Arachis hypogaea) and Mung Bean (Vigna radiata)
Source: Microbiol Spectr. 2022 Dec 8;11(1):e02096-22. doi: 10.1128/spectrum.02096-22 (PMC9927474; doi:10.1128/spectrum.02096-22)
Supplement: Supplemental file 1 — Fig. S1 to S8 and Table S3. Download spectrum.02096-22-s0001.pdf, PDF file, 1.3 MB [file spectrum.02096-22-s0001.pdf]

# Figure S1.

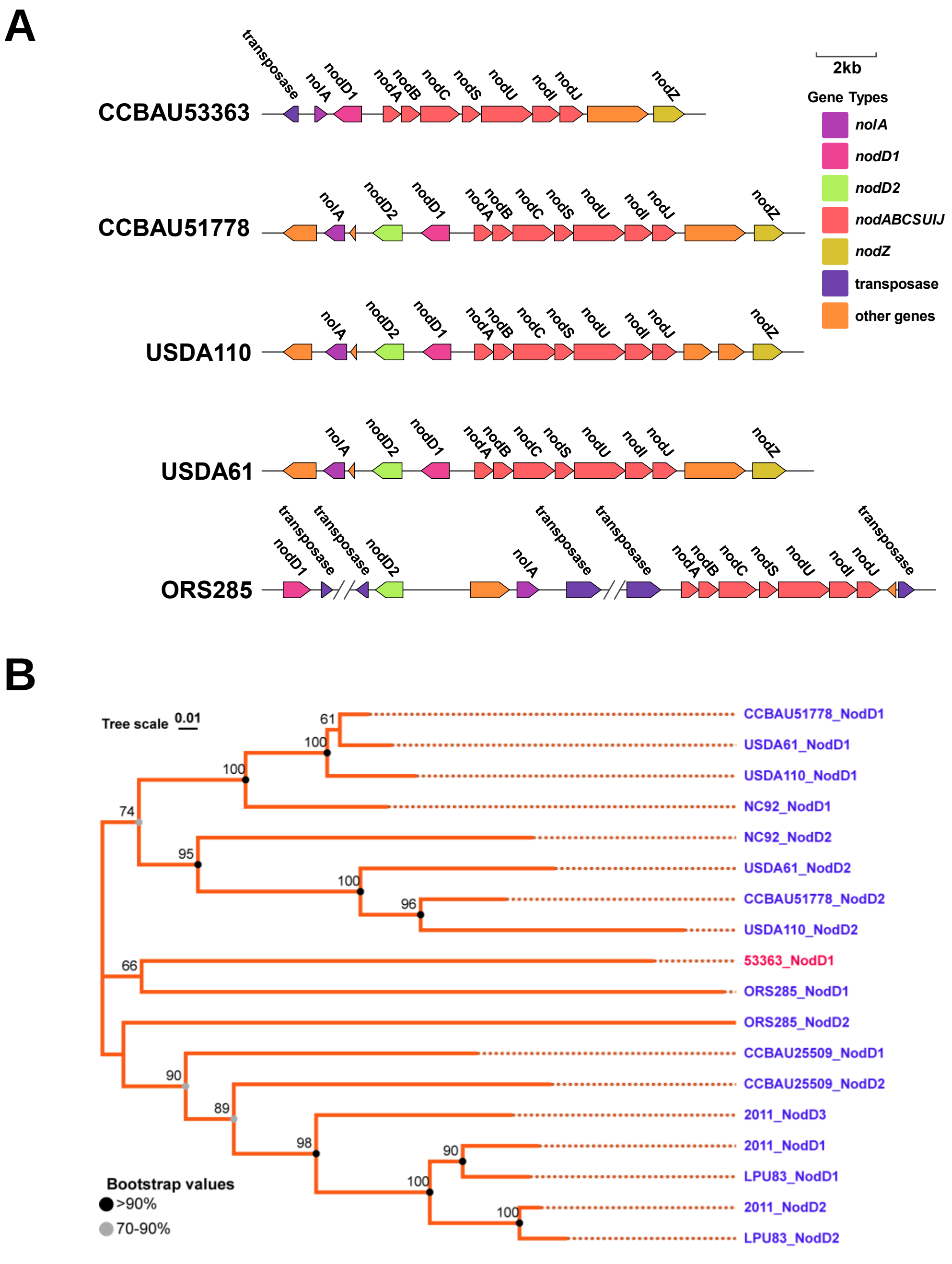


**Fig. S1. Schematic comparison of *nolA* and *nodD* genes relative to the *nod* operon in CCBAU53363 with those in other bradyrhizobia and phylogenic tree of NodD proteins.** (**A**) The presence of conserved *nodABCSUIJ* operon in the corresponding genome of the following bradyrhizobia: *B. zhanjiangense* CCBAU51778, *B. diazoefficiens* USDA110, *B. elkanii* USDA61 and *Bradyrhizobium* sp. ORS285 characterized by two copies of *nodD* and full-version of *nolA* is shown to be distinct from CCBAU53363 harboring only one *nodD* copy and a truncated *nolA* gene, and the gene orientation of the truncated *nolA* is opposed to *nodD1* in contrast to those in the other bradyrhizobia. (**B**) Neighbor-Joining phylogenic tree based on NodD proteins of representative rhizobial strains. The percentage of replicate trees in which the associated taxa clustered together in the bootstrap test (1000 replicates) are shown next to the branches. The evolutionary distances were computed using the JTT matrix-based method and are in the units of the number of amino acid substitutions per site. This analysis involved 18 amino acid sequences. Evolutionary analyses were conducted in MEGA X.

# Figure S2.

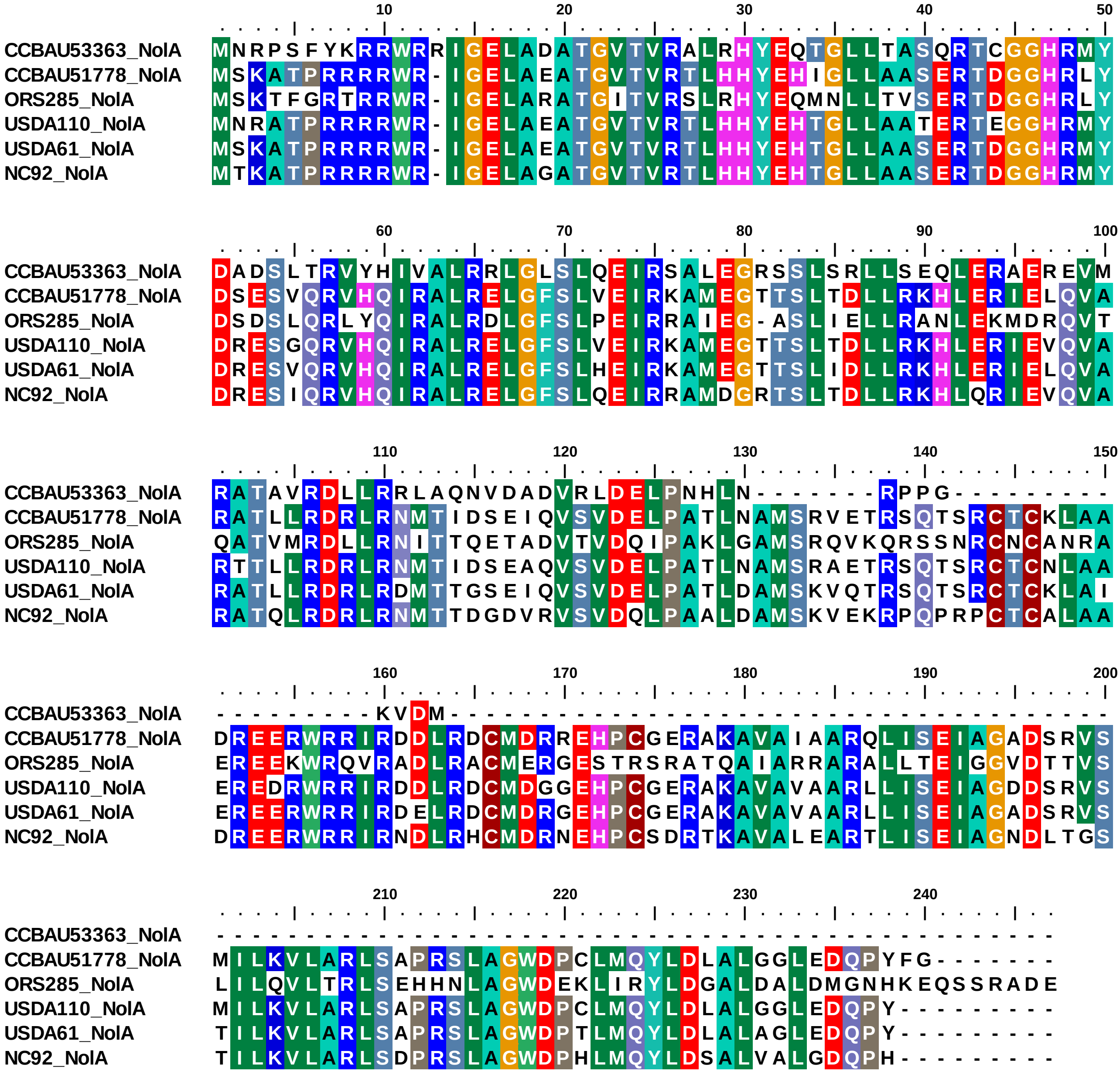


**Fig. S2. Sequence alignment of CCBAU53363 NolA protein with other bradyrhizobial NolA proteins.** The protein sequence of CCBAU53363 NolA was aligned with NolA proteins from the following bradyrhizobia: *B. diazoefficiens* USDA110, *B. elkanii* USDA61, *B. zhanjiangense* CCBAU51778 and *Bradyrhizobium* sp. ORS285 using MAGE X ~~(76)~~, the resulting file was further edited by BioEdit.

# Figure S3.

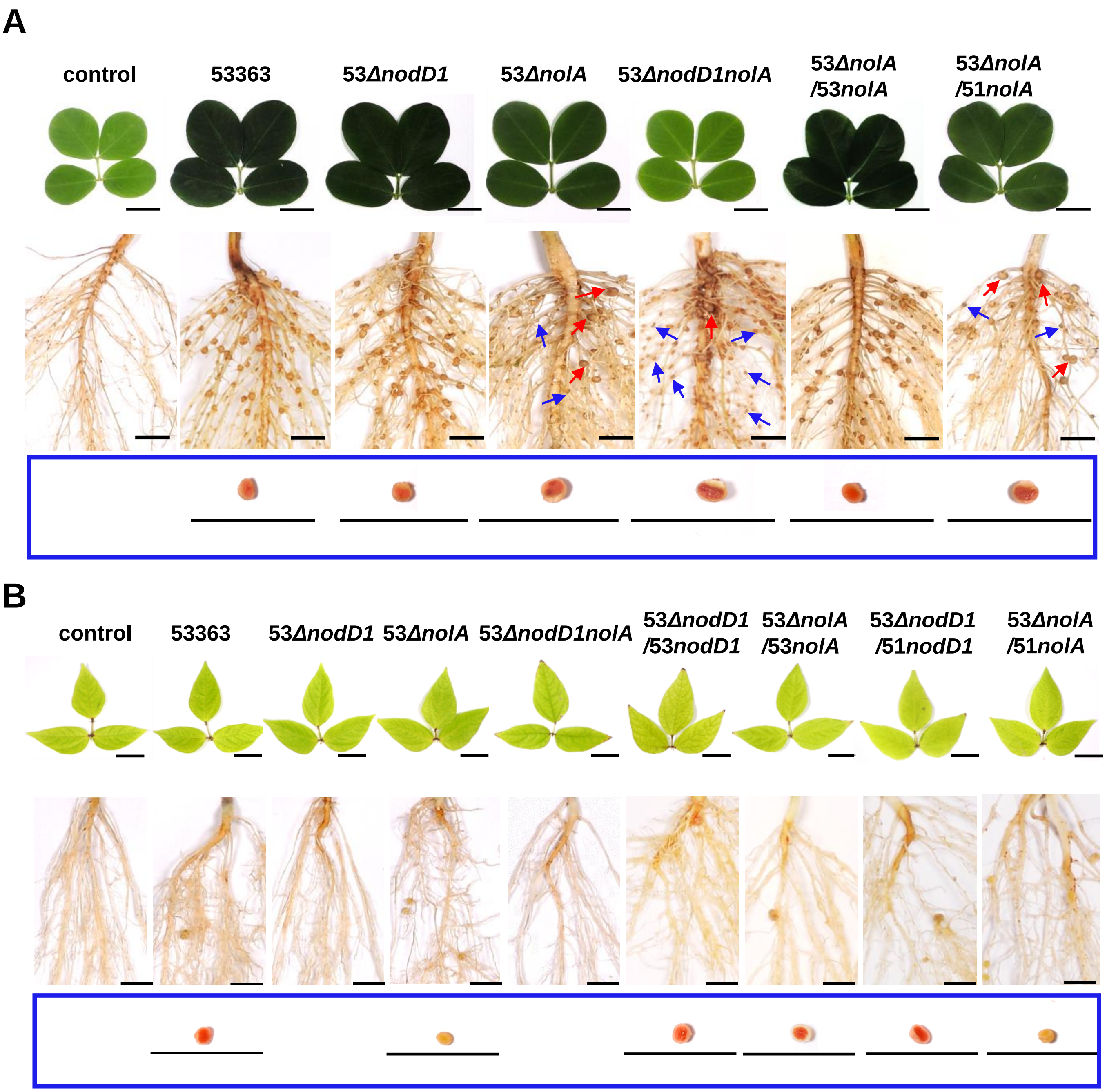


**Fig. S3. Symbiotic performance of CCBAU53363 mutants lacking *nolA* or/and *nodD1* on peanut and mung bean.** Leaves, roots and nodules of peanut (**A**) at 45 dpi or mung bean (**B**) at 30 dpi after inoculation with CCBAU53363 and its derivates. Images of peanut roots induced by the monogenic mutant 53Δ*nolA*, the polygenic mutant 53Δ*nodD1nolA*, and the heterogenous complementary strain 53Δ*nolA*/51*nolA* showed some nodules (red arrows) and bumps (blue arrows). Scale bars represent 1.5 cm for peanut and 1.0 cm for mung bean, respectively.

# Figure S4.

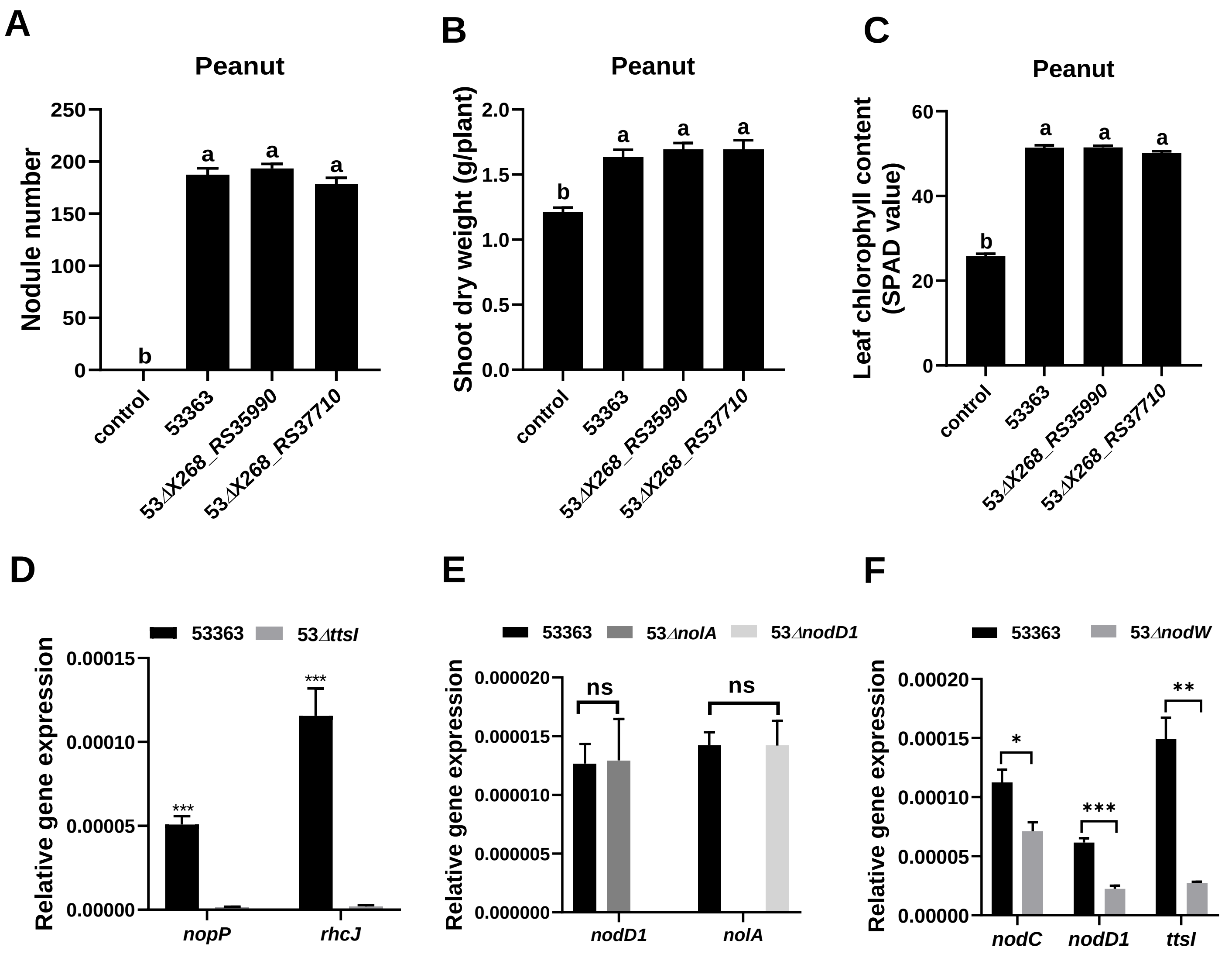


**Fig. S4. Characterization of representative genes putatively regulated by NolA for their roles in CCBAU53363 symbiosis with peanut and regulatory relationship verified by qRT-PCR.** (**A-C**) Number of infected nodules (**A**), shoot dry weight (**B**), leaf chlorophyll content (**C**) of peanut inoculated with the wild-type CCBAU53363 and its derivate strains: 53Δ*X268_RS37710* and 53Δ*X268_RS35990*. Values shown are means ± SEM of more than 23 plants scored from three independent experiments (7-9 replicate plants per treatment). Different lowercase letters indicate significant differences among means based on Duncan's test (α = 0.05). (**D-F**) qRT-PCR analysis of *rhcJ* and *nopP* transcription in wild-type CCBAU53363 and 53Δ*ttsI* (**D**), and transcription of *nolA* and *nodD1* in wild-type CCBAU53363 and corresponding mutants (53Δ*nodD1* or 53Δ*nolA*) (**E**), and the transcription of *nodC*, *nodD1* and *ttsI* genes in the wild-type CCBAU53363 and 53Δ*nodW* (**F**). All strains were induced by 1 µM genistein for 22 h. Results by means ± SEM from biological replicates in three independent experiments are shown (*t* test, ns (not significant), P > 0.05; *, P < 0.05; **, P < 0.01; ***, P < 0.001).

# Figure S5.

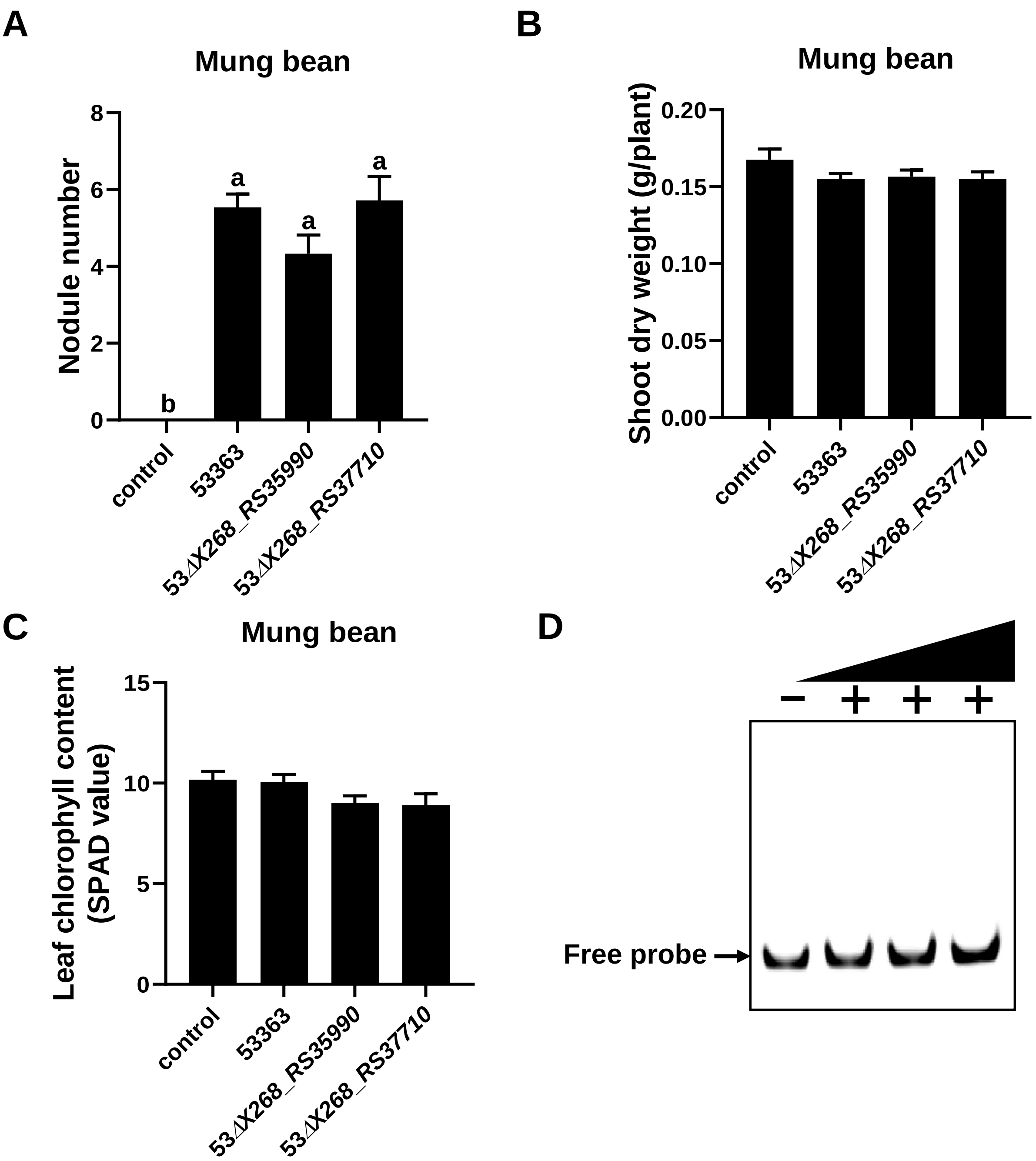


**Fig. S5. Characterization of representative genes putatively regulated by NolA for roles in CCBAU53363 symbiosis with mung bean and EMSA assay to determinate whether *nopP* was regulated by NolA directly. (A-C)** Number of infected nodules (**A**), shoot dry weight (**B**), leaf chlorophyll content (**C**) of mung bean inoculated with the wild-type CCBAU53363 and its derivate strains: 53Δ*X268_RS37710* and 53Δ*X268_RS35990*. Values shown are means ± SEM of more than 23 plants scored from three independent experiments (7-9 replicate plants per treatment). Different lowercase letters indicate significant differences among means based on Duncan's test (α = 0.05). (**D**) CY5-labeled promoter fragment of *nopP* gene was incubated with purified NolA (3.33 μM of maximum concentration). - and + indicate the ~~presence~~ and ~~absence~~ of NolA protein, respectively.

# Figure S6.

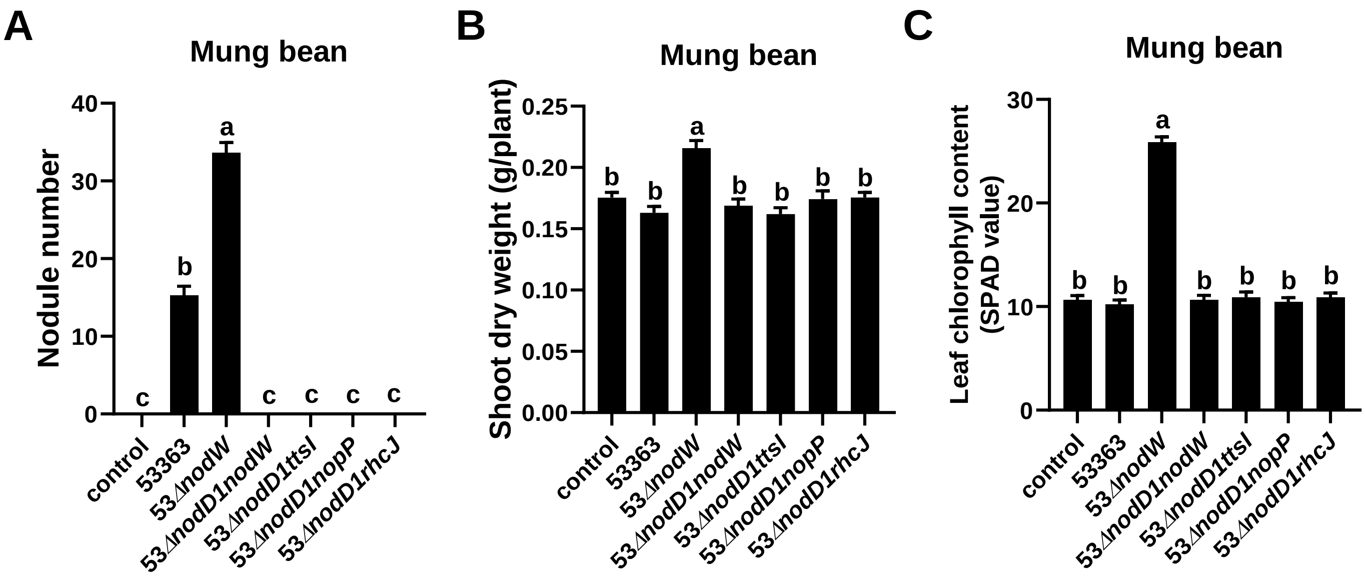


**Fig. S6. *nodW* was found to restrict CCBAU53363 to nodulate mung bean.** (**A**-**C**) Deletion of *nodW* in CCBAU53363 to enhance the symbiotic capability on mung bean. Number of infected nodules (**A**), shoot dry weight (**B**), leaf chlorophyll content (**C**) of mung bean inoculated with the wild-type CCBAU53363, 53Δ*nodW*, 53Δ*nodD1nodW* and mutants of 53Δ*nodD1* lacking different T3SS genes. Values shown are means ± SEM of more than 20 plants scored from three independent experiments (6-9 replicate plants per treatment). Different lowercase letters indicate significant differences among means based on Duncan's test (α = 0.05).

# Figure S7.

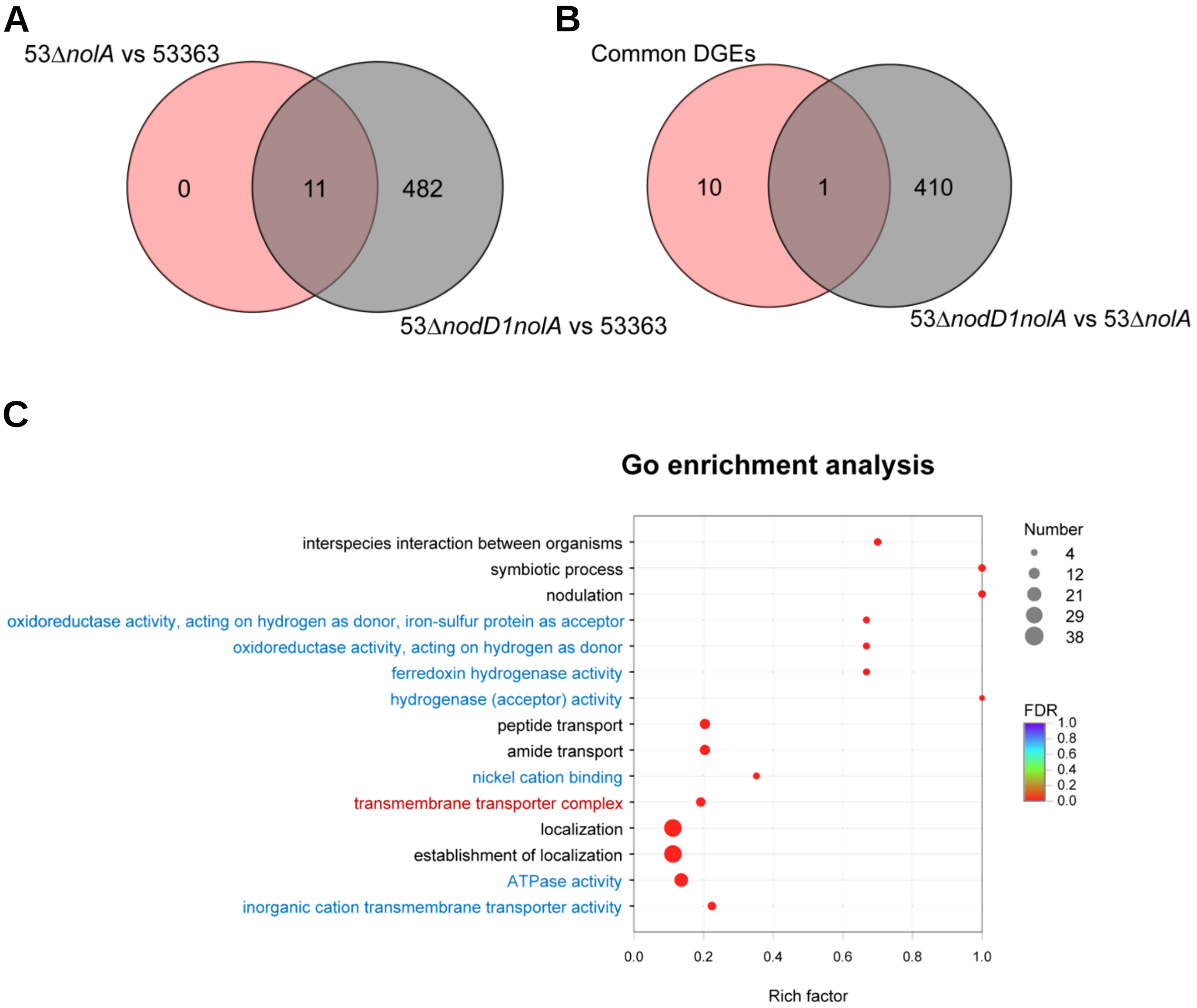


**Fig. S7. Venn diagram analysis and GO enrichment analysis of DEGs which were regulated by NolA-NodD1. (A)** Venn diagram analysis of DEGs in 53Δ*nolA* (53Δ*nolA* vs CCBAU53363) and those in 53Δ*nodD1nolA* (53Δ*nodD1nolA* vs CCBAU53363). (**B**) Venn diagram analysis of 11 shared DEGs and DEGs in ~~53Δ*nolA*~~ compared to 51Δ~~*nodD1*~~*nolA*. (**C**) GO enrichment analysis of 483 DEGs. The ordinate represents the GO term, and the black, red and blue fonts indicate that the GO term belongs to biological process, cellular component and molecular function, respectively. The abscissa is the rich factor (enrichment rate), which indicates the degree of enrichment. The larger value corresponds to higher degree of enrichment. The size of the dot represents the number of genes in the corresponding GO term, and the color of the dot represents the FDR range. FDR: P-value adjusted by multiple hypothesis testing (Significantly enriched, FDR < 0.05; Extremely significantly enriched, FDR < 0.01).

# Figure S8.

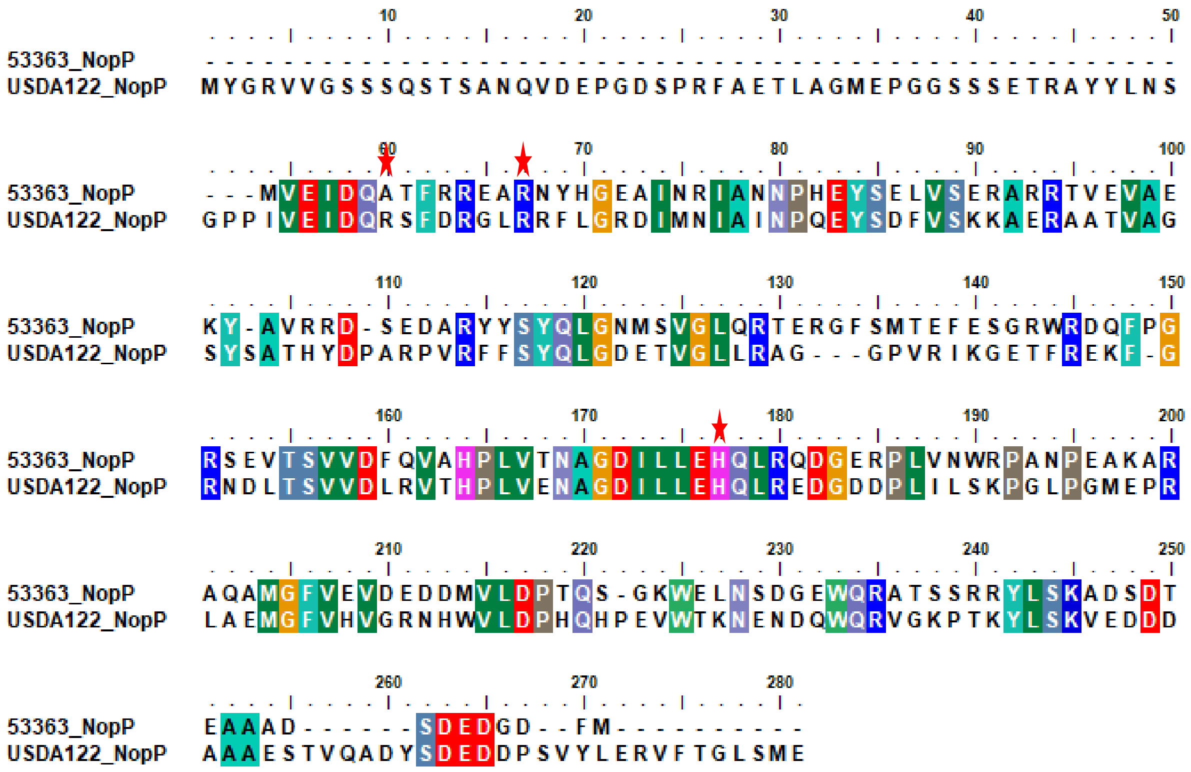


**Fig. S8. Sequence alignment of CCBAU53363 NopP protein with *B. diazoefficiens* USDA122 NopP protein.** The protein sequence of CCBAU53363 NopP was aligned with NopP protein from *B. diazoefficiens* USDA122 using MAGE X, the resulting file was further edited by BioEdit. Three critical amino acid residues (R60, R67 and H173) in USDA122 are indicated with red stars.

**Table S3. GO enrichment analysis of the new gene set with 483 DEGs.**

| GO ID | Function Description | Category | Gene Num | Ratio in DEGs | Ratio in Gom | FDR |
|---|---|---|---|---|---|---|
| GO:0044403 | symbiotic process | biological_process | 7 | 7/254 | 7/3666 | 0.0001 |
| GO:0009877 | nodulation | biological_process | 7 | 7/254 | 7/3666 | 0.0001 |
| GO:0044419 | interspecies interaction between organisms | biological_process | 7 | 7/254 | 10/3666 | 0.0000 |
| GO:0015833 | peptide transport | biological_process | 13 | 13/254 | 64/3666 | 0.0038 |
| GO:0042886 | amide transport | biological_process | 13 | 13/254 | 64/3666 | 0.0038 |
| GO:0022904 | respiratory electron transport chain | biological_process | 5 | 5/254 | 13/3666 | 0.0098 |
| GO:0006810 | transport | biological_process | 38 | 38/254 | 332/3666 | 0.0088 |
| GO:0051179 | localization | biological_process | 38 | 38/254 | 334/3666 | 0.0085 |
| GO:0051234 | establishment of localization | biological_process | 38 | 38/254 | 334/3666 | 0.0085 |
| GO:0022900 | electron transport chain | biological_process | 5 | 5/254 | 15/3666 | 0.0105 |
| GO:0055085 | transmembrane transport | biological_process | 26 | 26/254 | 209/3666 | 0.0109 |
| GO:0071941 | nitrogen cycle metabolic process | biological_process | 7 | 7/254 | 30/3666 | 0.0130 |
| GO:0071705 | nitrogen compound transport | biological_process | 15 | 15/254 | 103/3666 | 0.0143 |
| GO:0009399 | nitrogen fixation | biological_process | 5 | 5/254 | 21/3666 | 0.0271 |
| GO:0071702 | organic substance transport | biological_process | 15 | 15/254 | 113/3666 | 0.0274 |
| GO:0071692 | protein localization to extracellular region | biological_process | 5 | 5/254 | 25/3666 | 0.0389 |
| GO:0002790 | peptide secretion | biological_process | 5 | 5/254 | 25/3666 | 0.0389 |
| GO:0046903 | secretion | biological_process | 5 | 5/254 | 25/3666 | 0.0389 |
| GO:0009306 | protein secretion | biological_process | 5 | 5/254 | 25/3666 | 0.0389 |
| GO:0032940 | secretion by cell | biological_process | 5 | 5/254 | 25/3666 | 0.0389 |
| GO:0035592 | establishment of protein localization to extracellular region | biological_process | 5 | 5/254 | 25/3666 | 0.0389 |

Continued

| | | | | | | |
|---|---|---|---|---|---|---|
| GO:0140352 | export from cell | biological_process | 5 | 5/254 | 25/3666 | 0.0389 |
| GO:0030254 | protein secretion by the type III secretion system | biological_process | 2 | 2/254 | 4/3666 | 0.0358 |
| GO:0042026 | protein refolding | biological_process | 2 | 2/254 | 5/3666 | 0.0481 |
| GO:0043190 | ATP-binding cassette (ABC) transporter complex | cellular_component | 11 | 11/254 | 55/3666 | 0.0099 |
| GO:0098533 | ATPase dependent transmembrane transport complex | cellular_component | 11 | 11/254 | 55/3666 | 0.0099 |
| GO:0009375 | ferredoxin hydrogenase complex | cellular_component | 3 | 3/254 | 4/3666 | 0.0093 |
| GO:1902495 | transmembrane transporter complex | cellular_component | 11 | 11/254 | 57/3666 | 0.0079 |
| GO:0098797 | plasma membrane protein complex | cellular_component | 11 | 11/254 | 60/3666 | 0.0097 |
| GO:1990351 | transporter complex | cellular_component | 11 | 11/254 | 61/3666 | 0.0104 |
| GO:1990204 | oxidoreductase complex | cellular_component | 5 | 5/254 | 17/3666 | 0.0150 |
| GO:0098796 | membrane protein complex | cellular_component | 11 | 11/254 | 75/3666 | 0.0319 |
| GO:0005886 | plasma membrane | cellular_component | 29 | 29/254 | 274/3666 | 0.0328 |
| GO:0016020 | membrane | cellular_component | 34 | 34/254 | 347/3666 | 0.0413 |
| GO:0016612 | molybdenum-iron nitrogenase complex | cellular_component | 2 | 2/254 | 5/3666 | 0.0481 |
| GO:0016610 | nitrogenase complex | cellular_component | 2 | 2/254 | 5/3666 | 0.0481 |
| GO:0016021 | integral component of membrane | cellular_component | 80 | 80/254 | 953/3666 | 0.0497 |
| GO:0031224 | intrinsic component of membrane | cellular_component | 80 | 80/254 | 954/3666 | 0.0494 |
| GO:0016699 | oxidoreductase activity, acting on hydrogen as donor, iron-sulfur protein as acceptor | molecular_function | 6 | 6/254 | 9/3666 | 0.0002 |
| GO:0016695 | oxidoreductase activity, acting on hydrogen as donor | molecular_function | 6 | 6/254 | 9/3666 | 0.0002 |
| GO:0008901 | ferredoxin hydrogenase activity | molecular_function | 6 | 6/254 | 9/3666 | 0.0002 |
| GO:0033748 | hydrogenase (acceptor) activity | molecular_function | 4 | 4/254 | 4/3666 | 0.0004 |

Continued

| | | | | | | |
|---|---|---|---|---|---|---|
| GO:0016151 | nickel cation binding | molecular_function | 6 | 6/254 | 17/3666 | 0.0065 |
| GO:0019829 | ATPase-coupled cation transmembrane transporter activity | molecular_function | 4 | 4/254 | 8/3666 | 0.0088 |
| GO:0022890 | inorganic cation transmembrane transporter activity | molecular_function | 9 | 9/254 | 40/3666 | 0.0086 |
| GO:0016887 | ATPase activity | molecular_function | 23 | 23/254 | 167/3666 | 0.0086 |
| GO:0017111 | nucleoside-triphosphatase activity | molecular_function | 25 | 25/254 | 195/3666 | 0.0091 |
| GO:0015662 | ion transmembrane transporter activity, phosphorylative mechanism | molecular_function | 3 | 3/254 | 5/3666 | 0.0110 |
| GO:0015077 | monovalent inorganic cation transmembrane transporter activity | molecular_function | 7 | 7/254 | 30/3666 | 0.0130 |
| GO:0009055 | electron transfer activity | molecular_function | 13 | 13/254 | 82/3666 | 0.0126 |
| GO:0016462 | pyrophosphatase activity | molecular_function | 25 | 25/254 | 203/3666 | 0.0132 |
| GO:0008519 | ammonium transmembrane transporter activity | molecular_function | 2 | 2/254 | 2/3666 | 0.0147 |
| GO:0015112 | nitrate transmembrane transporter activity | molecular_function | 2 | 2/254 | 2/3666 | 0.0147 |
| GO:0008324 | cation transmembrane transporter activity | molecular_function | 9 | 9/254 | 48/3666 | 0.0140 |
| GO:0016818 | hydrolase activity, acting on acid anhydrides, in phosphorus-containing anhydrides | molecular_function | 25 | 25/254 | 208/3666 | 0.0175 |
| GO:0016817 | hydrolase activity, acting on acid anhydrides | molecular_function | 25 | 25/254 | 208/3666 | 0.0175 |
| GO:0004040 | amidase activity | molecular_function | 4 | 4/254 | 12/3666 | 0.0177 |
| GO:0043167 | ion binding | molecular_function | 79 | 79/254 | 883/3666 | 0.0188 |
| GO:0046914 | transition metal ion binding | molecular_function | 17 | 17/254 | 133/3666 | 0.0277 |
| GO:0008827 | cytochrome o ubiquinol oxidase activity | molecular_function | 2 | 2/254 | 3/3666 | 0.0278 |
| GO:0004553 | hydrolase activity, hydrolyzing O-glycosyl compounds | molecular_function | 3 | 3/254 | 8/3666 | 0.0279 |

Continued

| | | | | | | |
|---|---|---|---|---|---|---|
| GO:0015318 | inorganic molecular entity transmembrane transporter activity | molecular_function | 11 | 11/254 | 73/3666 | 0.0302 |
| GO:0016787 | hydrolase activity | molecular_function | 54 | 54/254 | 579/3666 | 0.0300 |
| GO:0016163 | nitrogenase activity | molecular_function | 3 | 3/254 | 9/3666 | 0.0339 |
| GO:0016732 | oxidoreductase activity, acting on iron-sulfur proteins as donors, dinitrogen as acceptor | molecular_function | 3 | 3/254 | 9/3666 | 0.0339 |
| GO:0043169 | cation binding | molecular_function | 39 | 39/254 | 400/3666 | 0.0338 |
| GO:0015075 | ion transmembrane transporter activity | molecular_function | 11 | 11/254 | 80/3666 | 0.0359 |
| GO:0046873 | metal ion transmembrane transporter activity | molecular_function | 4 | 4/254 | 17/3666 | 0.0362 |
| GO:0070401 | NADP+ binding | molecular_function | 2 | 2/254 | 4/3666 | 0.0358 |
| GO:0008556 | potassium transmembrane transporter activity, phosphorylative mechanism | molecular_function | 2 | 2/254 | 4/3666 | 0.0358 |
| GO:0046872 | metal ion binding | molecular_function | 38 | 38/254 | 392/3666 | 0.0356 |
| GO:0051538 | 3 iron, 4 sulfur cluster binding | molecular_function | 2 | 2/254 | 5/3666 | 0.0481 |
| GO:0018697 | carbonyl sulfide nitrogenase activity | molecular_function | 2 | 2/254 | 5/3666 | 0.0481 |
| GO:0030234 | enzyme regulator activity | molecular_function | 3 | 3/254 | 12/3666 | 0.0492 |
| GO:0016730 | oxidoreductase activity, acting on iron-sulfur proteins as donors | molecular_function | 3 | 3/254 | 12/3666 | 0.0492 |
| GO:0098772 | molecular function regulator | molecular_function | 3 | 3/254 | 12/3666 | 0.0492 |

Note: Gene Num is the number of DEGs annotated to this GO term; Ratio in DEGs is the ratio of the gene number in this GO term to the total number of DEGs with GO annotations; Ratio in Gom is the ratio of the gene number in this GO term in the whole genome to the total number of genes in the whole genome with GO annotations. FDR is the false discovery rate, which is obtained by correcting the p-value for significant differences.
